# MiR-146a-5p deficiency in extracellular vesicles of glioma-associated macrophages promotes epithelial-mesenchymal transition through the NF-κB signaling pathway

**DOI:** 10.1038/s41420-023-01492-0

**Published:** 2023-06-30

**Authors:** Chao Xu, Pan Wang, Haiyan Guo, Chuan Shao, Bin Liao, Sheng Gong, Yanghao Zhou, Bingjie Yang, Haotian Jiang, Gang Zhang, Nan Wu

**Affiliations:** 1 Chongqing Medical University, Chongqing, China; 2 Chongqing Institute of Green and Intelligent Technology, Chinese Academy of Sciences, Chongqing, China; 3 Chongqing School, University of Chinese Academy of Sciences, Chongqing, China; 4 Department of Neurosurgery, Chongqing General Hospital, Chongqing, China; 5 College of Life Sciences, University of Chinese Academy of Sciences, Beijing, China

**Keywords:** Cancer microenvironment, CNS cancer, Immune cell death, Cell invasion

## Abstract

Glioma-associated macrophages (GAMs) are pivotal chains in the tumor immune microenvironment (TIME). GAMs mostly display M2-like phenotypes with anti-inflammatory features related to the malignancy and progression of cancers. Extracellular vesicles derived from immunosuppressive GAMs (M2-EVs), the essential components of the TIME, greatly impact the malignant behavior of GBM cells. M1- or M2-EVs were isolated in vitro, and human GBM cell invasion and migration were reinforced under M2-EV treatment. Signatures of the epithelial-mesenchymal transition (EMT) were also enhanced by M2-EVs. Compared with M1-EVs, miR-146a-5p, considered the key factor in TIME regulation, was deficient in M2-EVs according to miRNA-sequencing. When the miR-146a-5p mimic was added, EMT signatures and the invasive and migratory abilities of GBM cells were correspondingly weakened. Public databases predicted the miRNA binding targets and interleukin 1 receptor-associated kinase 1 (IRAK1) and tumor necrosis factor receptor-associated factor 6 (TRAF6) were screened as miR-146a-5p binding genes. Bimolecular fluorescent complementation and coimmunoprecipitation confirmed interactions between TRAF6 and IRAK1. The correlation between TRAF6 and IRAK1 was evaluated with immunofluorescence (IF)-stained clinical glioma samples. The TRAF6-IRAK1 complex is the switch and the brake that modulates IKK complex phosphorylation and NF-κB pathway activation, as well as the EMT behaviors of GBM cells. Furthermore, a homograft nude mouse model was explored and mice transplanted with TRAF6/IRAK1-overexpressing glioma cells had shorter survival times while mice transplanted with glioma cells with miR-146a-5p overexpression or TRAF6/IRAK1 knockdown lived longer. This work indicated that in the TIME of GBM, the deficiency of miR-146a-5p in M2-EVs enhances tumor EMT through disinhibition of the TRAF6-IRAK1 complex and IKK-dependent NF-κB signaling pathway providing a novel therapeutic strategy targeting the TIME of GBM.

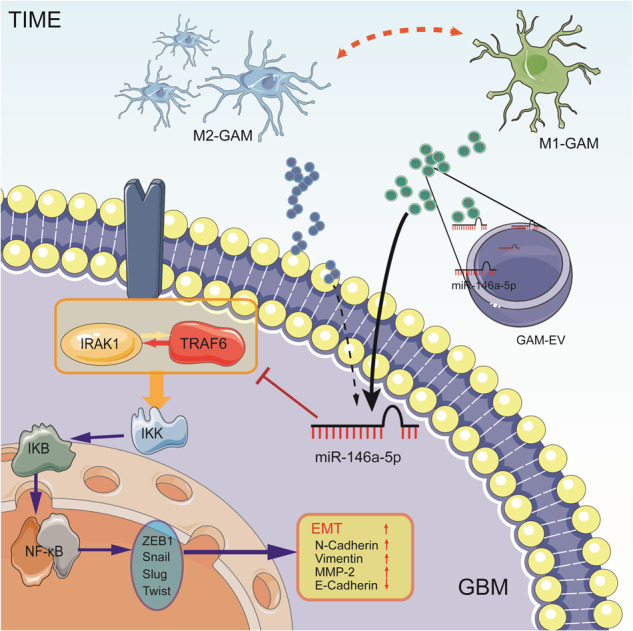

## Introduction

Glioblastoma (GBM) is a fatal cranial malignant tumor that accounts for almost 57% of all gliomas and exhibits a highly unfavorable outcome [1]. Currently, the recommended GBM therapeutic strategy is surgical resection followed by chemo- and radiotherapy [2]. Despite remarkable progress in targeting GBM, the overall prognosis remains poor [3]. Due to its multiple therapeutic resistance, uncontrollable growth, and unpredictable relapse events [4, 5]. The tumor immune microenvironment (TIME) comprises various immune cells and numerous soluble inflammatory mediators. It exhibits unique metabolic and immunological features that influence the malignant behaviors and development of GBM [6, 7]. Tumor progression is primarily determined by local immune responses linked with cancer cells [8]. However, it has not been easy to uncover the cellular interplay within the glioma TIME due to its complex and multidimensional communication. With the revelation of the connection between tumor-associated macrophages (TAMs) and tumor cells, TAMs have been recognized as one of the most critical components affecting tumor treatment. Nevertheless, due to the high temporospatial heterogeneity and hereditary plasticity, TAM-related immunotherapies have not achieved encouraging efficacy.

Glioma-associated macrophages/microglia (GAMs) account for over half of the GBM tumor bulk’s live cells [9]. Via a complex multidimensional regulatory network, GAMs can be polarized toward proinflammatory (M1-like) and anti-inflammatory (M2-like) phenotypes and thus exhibit antitumor or protumor effects, respectively. The M1 and M2 phenotypes are the extreme immune states of activated macrophages representing the common immunological features of infiltrating macrophages [10]. Compared with proinflammatory macrophages, GAMs show both immunostimulatory and suppressive features simultaneously, and promote gliomagenesis, continued tumor growth, and increased malignancy [11–13]. Extracellular vesicles (EVs) derived from GAMs are important components of the TIME and contain various cargos, including peptides, lipids, mRNAs, and microRNAs (miRNAs), which are crucial vehicles for communication between tumor cells and GAMs [14, 15]. Among these biologically active cargos, miRNAs are small but mighty [16] and greatly impact the progression and recurrence of GBM [17, 18].

In this work, based on open online databases, we confirmed that in GBM, immunosuppressive genes related to GAM were notably elevated and that the mRNA profile of GAMs more closely resembled that of the M2 phenotype. We focused on the differences in tumor cell behaviors caused by GAMs with different immunological characteristics and noticed that EVs derived from M2-like GAMs (M2-EVs) induced tumor cell invasion and migration. Comparisons of miRNAs in EVs showed that miR-146a-5p, which damages NF-κB activities and EMT behavior by binding to interleukin 1 receptor-associated kinase 1 (IRAK1) and tumor necrosis factor receptor-associated factor (TRAF6), was deficient in M2-EVs, suggesting that miR-146a-5p could be an encouraging GBM treatment candidate.

## Results

### Immunosuppressive GAM-associated genes are upregulated in GBM

**Fig. 1:**
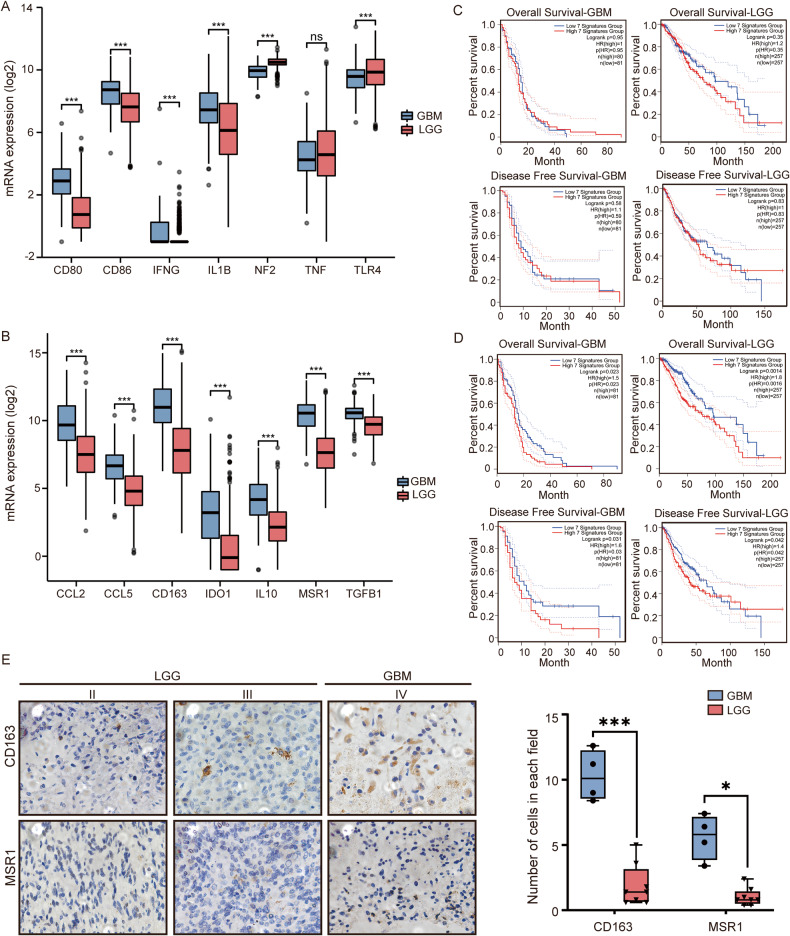
Immunosuppressive glioma-associated macrophage (GAMs)-related genes were upregulated in glioblastoma multiforme (GBM) compared with low-grade glioma (LGG). **A** The mRNA expression of the immunostimulatory genes CD80, CD86, IFNG, IL1B, NF2, TNF, and TLR4 in LGG and GBM in The Cancer Genome Atlas (TCGA) database was analyzed. **B** The mRNA expression of the immunosuppressive genes CCL2, CCL5, CD163, IDO1, IL10, MSR1, and TGFB in LGG and GBM in TCGA was analyzed. **C** The overall survival (OS) and disease-free survival (DFS) of GBM and LGG patients in TCGA with differential expression of CD80, CD86, IFNG, IL1B, NF2, TNF, and TLR4 was analyzed with GEPIA 2.0. **D** The OS and DFS of patients with GBM and LGG in TCGA with differential expression of CCL2, CCL5, CD163, IDO1, IL10, MSR1, and TGFB was analyzed with GEPIA 2.0. **E** The levels of TLR4, CD86, CD163, and MSR1 in GBM (*n* = *4*) and LGG (grade II and III gliomas, *n* = *8*) clinical samples were determined by immunohistochemical (IHC) staining and quantitative analysis. Scale bar = 50 μm. *p < 0.05, **p < 0.01, ***p < 0.001.

To explore the immune status of the infiltrated macrophages in GBM, we evaluated the differential expression of seven immunostimulatory GAM-related genes (TLR-4, IFNG, IL1B, TNF, NF2, CD80, and CD86) and seven immunosuppressive GAM-related genes (IDO, IL10, TGFB1, CCL2, CCL5, CD163, and MSR1) in patients with LGG and GBM and the relationships of these genes with survival in TCGA database (Fig. 1A, B). All immunosuppressive genes and five immunostimulatory genes were elevated in GBM patients compared with LGG patients. NF2 and TLR4 were downregulated in GBM. Overall survival (OS) and disease-free survival (DFS) were not influenced by the expression of immunostimulatory genes in these two groups (Fig. 1C). However, when the seven immunosuppressive genes were highly expressed, the OS and DFS times were significantly decreased. (Fig. 1D). To verify the findings in clinical cases, CD163 and MSR1 expression was evaluated by immunohistochemical (IHC) in 12 human glioma specimens. The numbers of CD163- and MSR1-positive cells were higher in the GBM slices than in grade II and grade III glioma specimens (Fig. 1E). These findings indicated that infiltrating GAMs exhibit more immunosuppressive features in high-grade gliomas and that high expression of immunosuppressive genes is tightly related to GBM progression and unfavorable outcomes.

### Coculture with M2-EVs enhanced U87MG and A172 cell invasion and migration

**Fig. 2:**
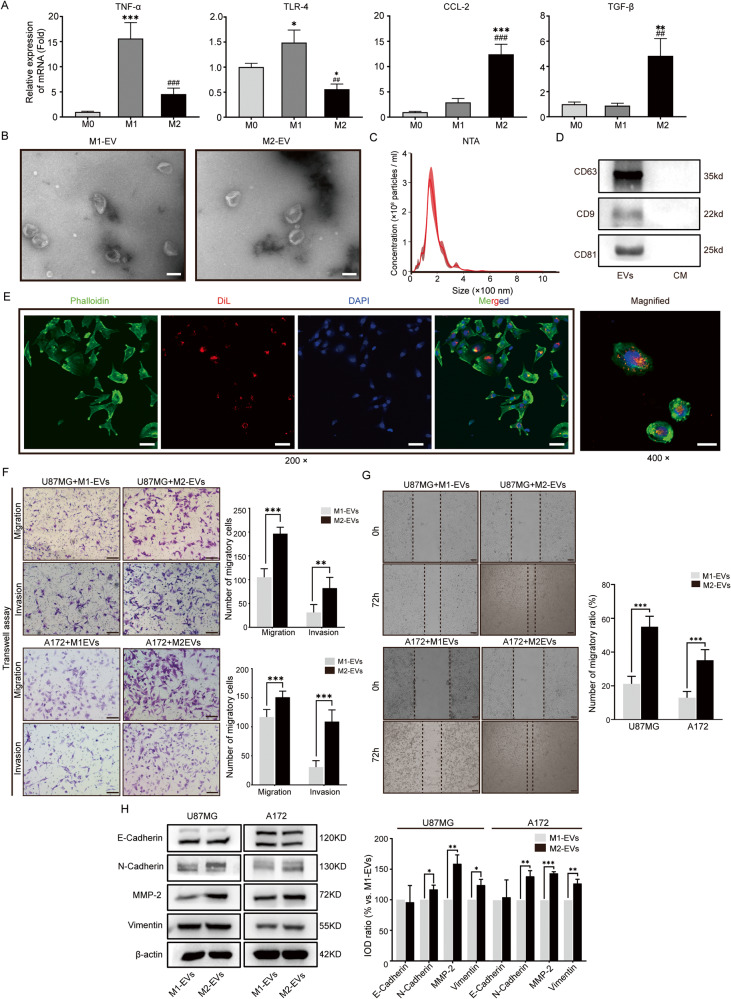
Immunostimulatory (M1-like) and immunosuppressive (M2-like) GAMs were established with THP-1 cells in vitro, and the extracellular vesicles (EVs) derived from these cells were isolated. **A** RT‒PCR was performed to measure the expression of immunostimulatory (TNF-α and TLR-4) and immunosuppressive (CCL-2 and TGF-β) markers in M2-GAMs generated by stimulation with GM-CSF and IL-4/IL-13, respectively, in vitro. M0 represents inactivated macrophages (M0). *n* = 3. **B** EVs extracted from M1-like and M2-like GAMs were observed by transmission electron microscopy. Both M1-EVs and M2-EVs exhibited bilayer membrane biconvex disc-shaped structures. Scale bar = 200 nm. **C** NTA showed that the diameters of EVs extracted from M1-like and M2-like GAMs were between 30 and 150 nm. *n* = 3. **D** Western blotting assays were conducted to determine the signature markers of EVs. CD63, CD9, and CD81 were significantly expressed in isolated EVs compared with conditioned medium (CM). **E** U87MG cells stained with phalloidin (green) and Hoechst (blue) were incubated with Dil-labeled M1-EVs and M2-EVs (red) for 12 h (left panel, scale bar = 50 μm). EVs were internalized into U87MG cells. The right panel shows the magnified image (scale bar = 50 μm). Compared with M1-EVs, M2-EVs enhanced U87MG and A172 cell invasion and migration. **F** Transwell assays and quantitative analysis were used to evaluate the invasive and migratory abilities of U87MG cells and A172 cells cocultured with M1-EVs or M2-EVs for 24 h. Scale bar = 200 μm. **G** To evaluate the migratory ability of U87MG cells and A172 cells cocultured with M1-EVs or M2-EVs over a 72 h-period, a wound healing assay and quantitative analysis were performed. Scale bar = 200 μm. **H** Western blotting was used to analyze the protein levels of E-cadherin, N-cadherin, vimentin, and MMP-2 in U87MG and A172 cells cocultured with M1-EVs and M2-EVs for 72 h. *n* = 3, *p < 0.05, **p < 0.01, ***p < 0.001; ##*p* < 0.01 and ###*p* < 0.001.

EVs are the most critical vesicles for communication between GAMs and GBM cells and are indispensable components of the TIME. We found that immunosuppressive genes were upregulated in GBM, and M2-like GAMs were established to simulate the immunosuppressive characteristics of GAMs, while M1-like GAMs were established as a control. To verify that EVs derived from immunosuppressed GAMs are the main contributor to GBM malignant behaviors, THP-1 cells simulated immunostimulatory (M1-like) and immunosuppressive (M2-like) GAM models in vitro under GM-CSF or IL-4/IL-13 stimulation. Real-time polymerase chain reaction (RT‒PCR) was conducted to evaluate the mRNA levels of TNF-α, TLR-4, CCL-2, and TGF-β to validate the immunological features of M1- and M2-like GAMs. TNF-α and TLR-4 were upregulated in M1-like GAMs and downregulated in M2-like GAMs. In contrast, CCL-2 and TGF-β were downregulated in M1-like GAMs and upregulated in M2-like GAMs (Fig. 2A), suggesting that M1-like GAMs were in an immunostimulatory state and M2-like GAMs were in an immunosuppressive state. EVs derived from M1- or M2-like GAMs (M1- or M2-EVs, respectively) were isolated and found to have a 30–150 nm-diameter bilayer membrane biconvex disc-shaped structure by TEM (Fig. 2B) and nanoparticle tracking analysis (NTA; Fig. 2C). The specific markers CD63, CD9, and CD81 were detected in EVs and conditioned medium by western blotting (Fig. 2D), which ensured the purity of the EVs. After labeling with Dil, M1-EVs and M2-EVs were cocultured with U87MG cells whose cytoskeleton was stained with phalloidin, and red fluorescence was observed in the cytoplasm (Fig. 2E). Collectively, these findings demonstrated that M1-EVs and M2-EVs were successfully extracted from the medium supernatant and were able to be internalized into GBM cells.

Subsequently, the EMT behaviors of GBM cells were assessed under coculture with M1- and M2-EVs. Transwell (Fig. 2F) and wound healing assays (Fig. 2G) were conducted, and both U87MG and A172 cells cocultured with M2-EVs were more invasive than those cocultured with M1-EVs and displayed rapid wound closure within 72 h. Markers of EMT, including N-cadherin, MMP-2, and vimentin, were evaluated by Western blotting (Fig. 2H). N-cadherin, MMP-2, and vimentin were expressed at high levels in M2-EV-treated U87MG and A172 cells compared with cells treated with M1-EVs. In combination, our work suggested that compared with M1-EVs, M2-EVs increased GBM cell migratory and invasive abilities promoting EMT in these cells in vitro.

### MiR-146a-5p is deficient in M2-EVs

**Fig. 3:**
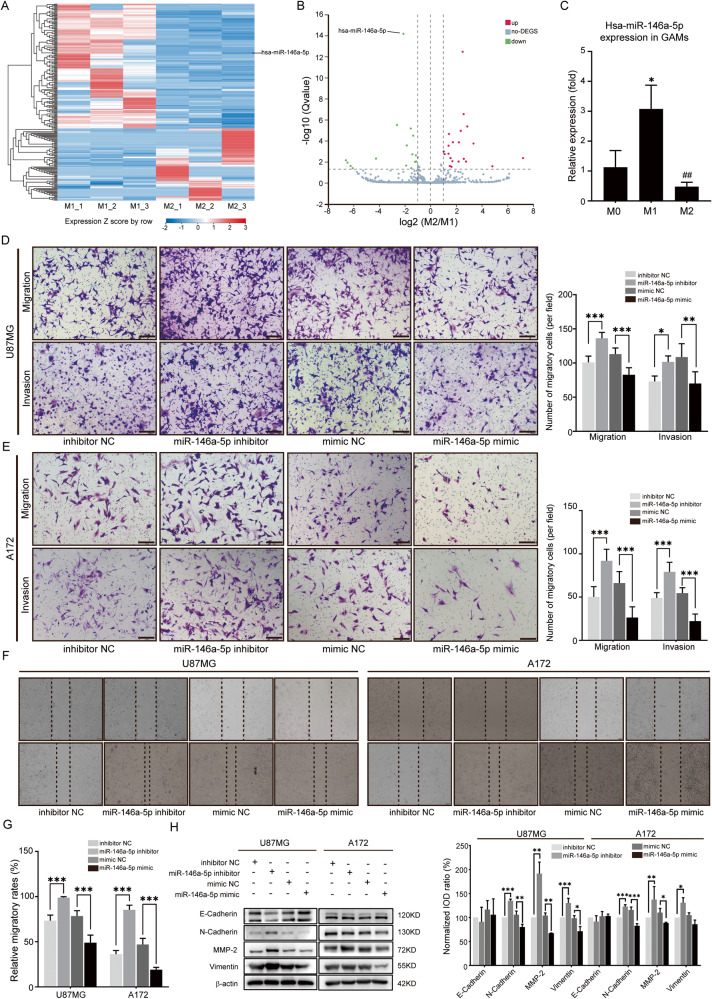
Compared to M1-EVs, miR-146a-5p was deficient in M2-EVs and enhanced U87MG and A172 cell invasion and migration behaviors. **A** The heatmap showed the differential expression of miRNAs between M1- and M2-EVs according to miRNA-sequencing. **B** The volcano graph plot showed the relationship between the fold change and the significance of differentially expressed miRNAs of M2-EVs compared to M1-EVs. **C** The miR-146a-5p level of M0, M1-like GAMs, and M2-like GAMs was evaluated by RT‒PCR. MiR-146a-5p was significantly increased in M1-like GAMs compared with M0 and M2-like GAMs. **D**, **E** Transwell assays and quantitative analysis were performed to evaluate the migratory and invasive abilities of U87MG cells and A172 cells transfected with the miR-146a-5p mimic, miR-146a-5p inhibitor or the corresponding negative control sequence (NC) for 24 h. **F**, **G** Wound healing assays and quantitative analysis were used to evaluate the migration ability of U87MG cells and A172 cells transfected with the miR-146a-5p mimic, miR-146a-5p inhibitor, mimic NC, or inhibitor NC for 24 h. **H** Western blotting analysis of the expression of E-cadherin, N-cadherin, MMP-2, and vimentin in U87MG and A172 cells transfected with the miR-146a-5p mimic, miR-146a-5p inhibitor, mimic NC, or inhibitor NC. *p < 0.05, **p < 0.01, ***p < 0.001.

In EV-dependent biological regulation, miRNAs are the prominent cargo and exert substantial impacts [19]. To determine the specific compositions and underlying mechanisms that lead to the functional differences between the effects of M1-EVs and M2-EVs on GBM cells, MiRNA sequencing (miRNA-Seq) was performed. The differential expression of miRNAs derived from M1- and M2-EVs was analyzed with DEGseq. There were 237 miRNAs with the most marked differences (Fig. 3A and Supplementary Table S1), and miR-146a-5p was selected (Fig. 3B) as a candidate. MiR-146a-5p levels among M0, M1-, and M2-like GAMs were estimated with RT-PCR and were higher in M1-like GAMs than in M2-like GAMs (Fig. 3C), confirming that miR-146a-5p was lower in M2-like GAMs.

### Exosomal miR-146a-5p promoted EMT in GBM cells

To verify whether malignant EMT behaviors are influenced by miR-146a-5p derived from GAM-EVs, migratory and invasive behaviors and the expression of EMT markers were evaluated in GBM cells. The miR-146a-5p mimic and inhibitor and their negative control (NC) were transfected into U87MG and A172 cells. The miR-146a-5p mimic reduced their migration and invasion in Transwell assays of U87MG and A172 cells, and decreased the wound healing ability, and cells with miR-146a-5p inhibitors were more capable of migrating and invasive (Fig. 3D–G). Furthermore, the levels of N-cadherin, MMP-2, and vimentin were significantly elevated when the miR-146a-5p inhibitor was applied to the cells. When these cells were treated with the miR-146a-5p mimic, the levels of N-cadherin and MMP-2 were markedly decreased (Fig. 3H).

### MiR-146a-5p binds to IRAK1 and TRAF6

**Fig. 4:**
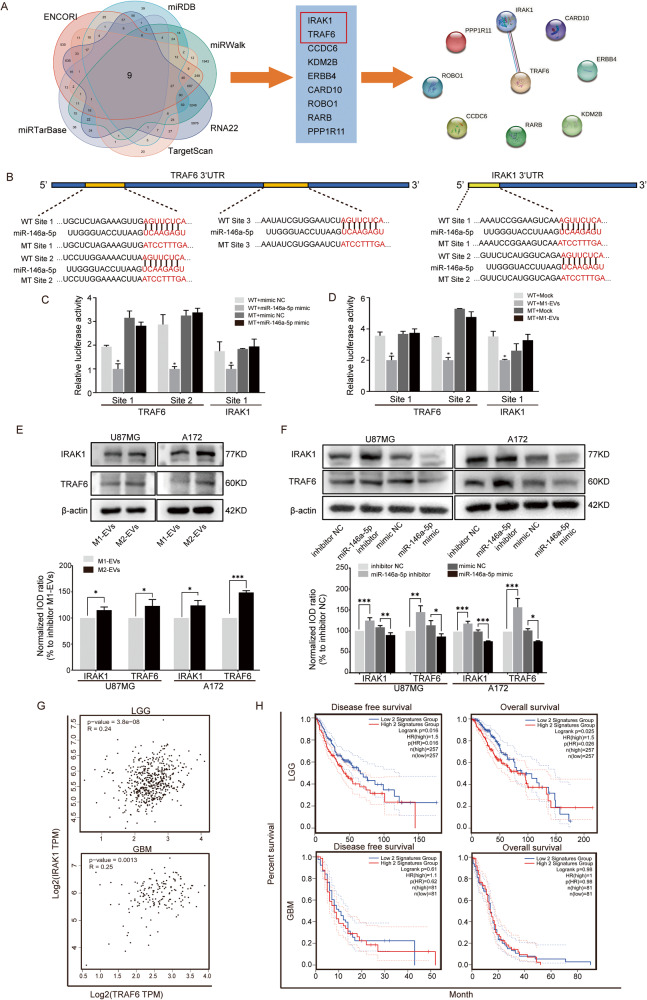
TRAF6 and IRAK1 are the miR-146a-5p target genes in GBM. **A** Venn diagram (left panel) showed the intersection of predicted binding genes of miR-146a-5p obtained from 6 online public databases. Nine genes (middle panel) were predicted in all six databases. Association analysis of the proteins encoded by these 9 genes performed with STRING (right panel) showed a strong connection between TRAF6 and IRAK1. **B** Schematic representation of the TRAF6 3′UTR and IRAK1 3′UTR. MT sequences were generated at the 3′UTR binding sites of miR-146a-5p according to the prediction. **C** Relative luciferase activity was determined in U87MG cells that were cotransfected with the miR-146a-5p mimic or mimic NC and the WT-3′UTR or MT-3′UTR of TRAF6 or IRAK1. **D** Relative luciferase activity was determined in U87MG cells transfected with the WT-3′UTR or MT-3′UTR of TRAF6 or IRAK1 for 24 h and then cultured with M1-EVs or mock. **E** Western blotting measured the expression levels of TRAF6 and IRAK1 in U87MG and A172 cells cocultured with M1-EVs or M2-EVs**. F** Expression of TRAF6 and IRAK1 in U87MG and A172 cells transfected with the miR-146a-5p mimic, miR-146a-5p inhibitor, mimic NC, or inhibitor NC. **G** The correlation of TRAF6 and IRAK1 mRNA expression in patients with LGG and GBM in TCGA was estimated, and the mRNA expression level of TRAF6 was positively correlated with that of IRAK1 in GBM and LGG. **H** The DFS and OS of glioma patients with different TRAF6/IRAK1 expressions were analyzed in TCGA database. Both DFS and OS of LGG patients with higher TRAF6/IRAK1 expression levels were shorter than those with lower expression levels. Both DFS and OS of GBM patients did not obtain significant benefit.

To clarify the potential downstream mechanisms of miR-146a-5p, six public online databases were used to predict its binding genes. Nine genes were then considered potential candidates. The relationships of these nine genes and their encoded proteins were analyzed with STRING, and it was found that TRAF6 and IRAK1 had stronger connections (Fig. 4A). According to the numbers of binding sites for miR-146a-5p predicted with TargetScan, TRAF6 has three conserved sites, and IRAK1 has two conserved sites. Therefore, IRAK1 and TRAF6 were selected as promising targets. Then, dual-luciferase reporter assays were executed to test whether miR-146a-5p was directly bound to these genes. TRAF6 and IRAK1 WT and MT 3′UTR-driven pmirGLO luciferase vectors were constructed (Fig. 4B), and the miR-146a-5p mimic was cotransfected into U87MG cells along with these vectors. The miR-146a-5p mimic inhibited the relative luciferase activity of these WT 3′UTRs compared to the control. In contrast, the luciferase activity of these MT 3′UTRs was not influenced by the miR-146a-5p mimic (Fig. 4C). Furthermore, M1-EVs were added to U87MG cells transfected with TRAF6 and IRAK1 WT and MT 3′UTR-driven pmirGLO luciferase vectors alone. The results showed that M1-EVs inhibited the luciferase activities of WT 3′UTR-driven luciferase (Fig. 4D). To detect the impact of M1-EVs and M2-EVs on the expression of TRAF6 and IRAK1, these EVs were added to U87MG and A172 cells. The expression level was detected by western blotting and showed that M2-EVs upregulated TRAF6 and IRAK1 compared with M1-EVs (Fig. 4E). Then, the miR-146a-5p mimic or inhibitor was transfected into U87MG and A172 cells and the mimic reduced the levels of TRAF6 and IRAK1, while the inhibitor increased their levels (Fig. 4F).

Furthermore, to clarify the expression of these two genes in glioma, we retrieved the data in TCGA and found that compared with LGG, the expression of IRAK1 and TRAF6 was upregulated in GBM (Supplementary Fig. S1). Both LGG and GBM express TRAF6 positively correlated with IRAK1 (Fig. 4G and Supplementary Fig. S2A) at the transcriptional level. We used TRAF6 and IRAK1 as a gene set and evaluated its effect on the survival time of glioma patients in TCGA. The LGG patients received longer survival times when they expressed lower levels of TRAF6 and IRAK1 but GBM patients did not obtain a remarkable benefit (Fig. 4H and Supplementary Fig. S2B), which might be caused by a large number of infiltrating M2-like GAMs rather than M1-like GAMs. These results confirmed that TRAF6 and IRAK1 are the targets of miR-146a-5p. Low IRAK1 levels increased the OS of LGG and overall glioma patients (Supplementary Fig. S2C) indicating that IRAK1 might be the direct effector modulating the progression of glioma.

Since the miRNA data of GBM patients were not available in TCGA database, we analyzed the correlation of miR-146a-5p vs. TRAF6 and vs. IRAK1 in the Chinese Glioma Genome Atlas (CGGA) database. However, the expression of miR-146a-5p was not negatively correlated with TRAF6 and IRAK1 in either LGG or GBM patients (Supplementary Fig. S3A), which seemed inconsistent with the changes we found in vitro. In addition, the OS of patients with different miR-146a-5p levels did not show a significant difference despite the longer survival time tendency presented in the high miR-146a-5p group (Supplementary Fig. S3B). We deduced that the intratumoral heterogeneity of glioma might lead to such inconsistencies of bulk RNA sequencing.

### TRAF6-IRAK1 complex was the regulatory target of miR-146a-5p

**Fig. 5:**
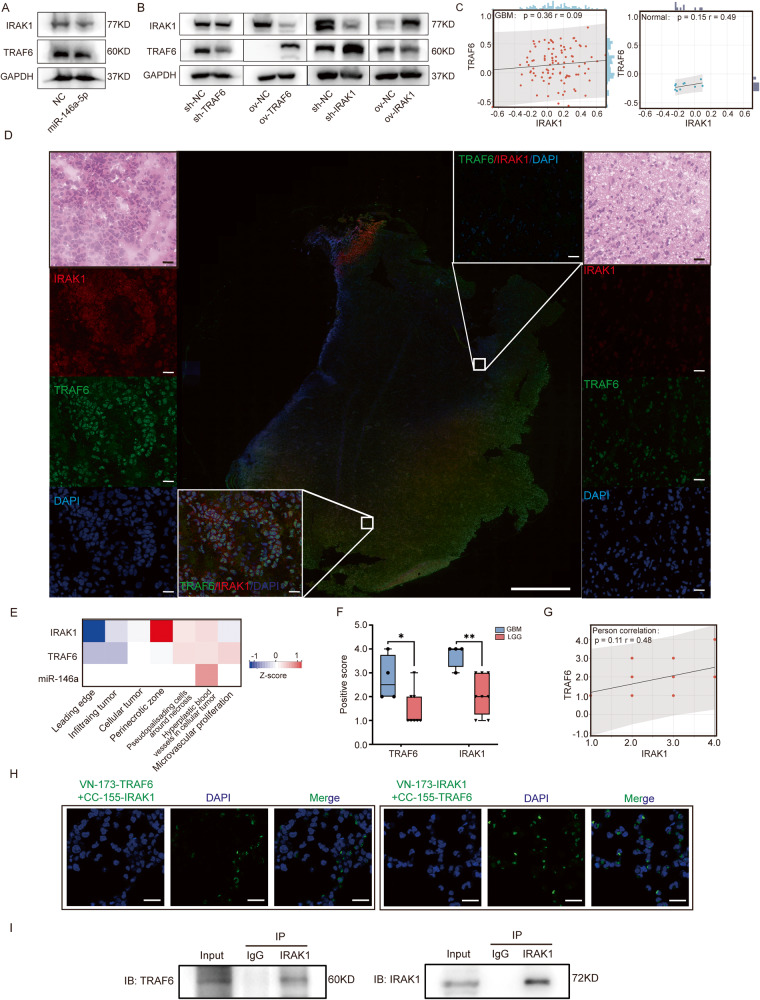
Complicated interactions exist between TRAF6 and IRAK1. **A**, **B** Western blotting was used to detected the expression of TRAF6 and IRAK1 at protein levels in U87MG cells with miR-146a-5p overexpression, and TRAF6 or IRAK1 knockdown, and TRAF6 or IRAK1 overexpression. **C** The expression correlations of TRAF6 and IRAK1 at protein levels in GBM (*n* = 99) and normal brain tissue (*n* = 10) were analyzed in CPTAC databases. **D** Slides derived from two GBM samples were evaluated with TRAF6 (green) and IRAK1 (red) dual-labeled immunofluorescence (IF). H&E staining was used to evaluate the pathological features and to identify specific areas (adjacent area not the same zone). Left panel showed the microvascular proliferation area and right panel showed the leading edge (scale bar = 20 μm). The scale bar of the merged panoramic image is 2000 μm. **E** The expressions of TRAF6, IRAK1, and miR-146a at mRNA levels in different GBM areas were analyzed with Ivy GAP database. **F** The levels of TRAF6 and IRAK1 in GBM (*n* = 4) and LGG (grade II and III gliomas, *n* = 8) clinical samples were determined by IF staining and positive scores were of each sample based on the fluorescence intensities and numbers of positive cell. The positive scores of TRAF6 and IRAK1 in GBM were remarkably higher than those in LGG. **G** The Person correlation was used to analyze the correlation of TRAF6 and IRAK1 expressions in clinical samples at protein levels with the positive scores. **H** BiFC was used to estimate the spatial formation of the TRAF6-IRAK1 complex. When pBiFC-VN173-TRAF6 and pBiFC-CC155-IRAK1 or pBiFC-VN173-IRAK1 and pBiFC-CC155-TRAF6 were cotransfected into U87MG cells, green fluorescence was detected in the cytoplasm. Scale bar = 50 µm. **I** Coimmunoprecipitation (CoIP) was performed to evaluate interactions between TRAF6 and IRAK1 at the protein level. The anti-IRAK1 antibody immunoblotted (IB) the TRAF6 that immunoprecipitated (IP) from total protein, while the IRAK1 IP from cell lysates and IB with an anti-TRAF6 antibody.

It has been reported that complicated posttranslational modifications (PTM) exist between TRAF6 and IRAK1. The correlation between these two genes at the protein level was evaluated. Cell lines with TRAF6 and IRAK1 being knockdown by shRNA were established, and cell lines with overexpression of these two genes and miR-146a-5p were also established at the same time. Overexpression of miR-146a-5p decreased the protein levels of TRAF6 and IRAK1 (Fig. 5A). Obviously, when TRAF6 was overexpressed, IRAK1 was significantly degraded and when IRAK1 was overexpressed, TRAF6 was inhibited. However, when TRAF6 was knocked down, we detected lightly decreased IRAK1, and when IRAK1 was knocked down, the expression of TRAF6 was remarkably increased (Fig. 5B). There was no significant change at the mRNA level (Supplementary Fig. 4A). GBM data from the Clinical Proteomic Tumor Analysis Consortium (CPTAC) were analyzed, and we found that the protein expression levels of these two molecules were highly expressed in GBM and also positively correlated despite insignificant differences (Fig. 5C and Supplementary Fig. 4B). The protein levels of TRAF6 and IRAK1 were estimated with IF-stained glioma samples. Two GBM samples were detected with double-labeled IF and the distribution of positive cells presented specific spatial features (Fig. 5D). TRAF6 was expressed at high levels in the cellular tumor zone and pseudopalisading cells around necrosis while in the microvascular proliferation zone and cellular tumor zone, IRAK1 was expressed at high levels. We referred to RNA-seq data in the Ivy Glioblastoma Atlas Project (Ivy GAP) and found that there were differences between the expression of these two genes in different areas (Fig. 5E and Supplementary Fig. 4C). Then, the 8 LGG samples and 4 GBM samples were stained with TRAF6 or IRAK1 at the continuously adjacent position, and the positive scores (defined by the fluorescence intensities and ratio of positive cells) of TRAF6 and IRAK1 in the GBM group were higher than those in the LGG group. Meanwhile, the positive scores of TRAF6 and IRAK1 were positively correlated (Fig. 5F, G and Supplementary Fig. S4D).

We noticed that in some area, TRAF6 and IRAK1 were able to be co-localized (Fig. 5E). It was reported that TRAF6-IRAK1-Pellino-1 complex mediated the activation of downstream pathways [20]. Therefore, we inferred that miR-146a-5p in M1-EVs exerts regulatory effects by targeting the TRAF6-IRAK1 complex. Bimolecular fluorescence complementation (BiFC) and coimmunoprecipitation (Co-IP) assays were performed to validate. The BiFC assay showed that when either pBiFC-VN173-TRAF6 and pBiFC-CC155-IRAK1 or pBiFC-VN173-IRAK1 and pBiFC-CC155-TRAF6 were cotransfected into U87MG cells, green fluorescence was detected in the cytoplasm (Fig. 5H). When pBiFC-VN173-TRAF6, pBiFC-VN173-IRAK1, pBiFC-CC155-TRAF6, and pBiFC-CC155-IRAK1 were transfected alone, fluorescence signals were rarely observed (Supplementary Fig. S5), indicating that there was spatial interaction between them. Co-IP showed that IRAK1 could be detected in the TRAF6 immunoprecipitate and that TRAF6 could be detected in the IRAK1 immunoprecipitate by IB (Fig. 5I), confirming that TRAF6 and IRAK1 interact with each other and form a complex and we established dual knockdown and dual overexpressing cell lines.

### MiR-146a-5p mitigates EMT in GBM through the IKKγ-dependent NF-κB signaling pathway

**Fig. 6:**
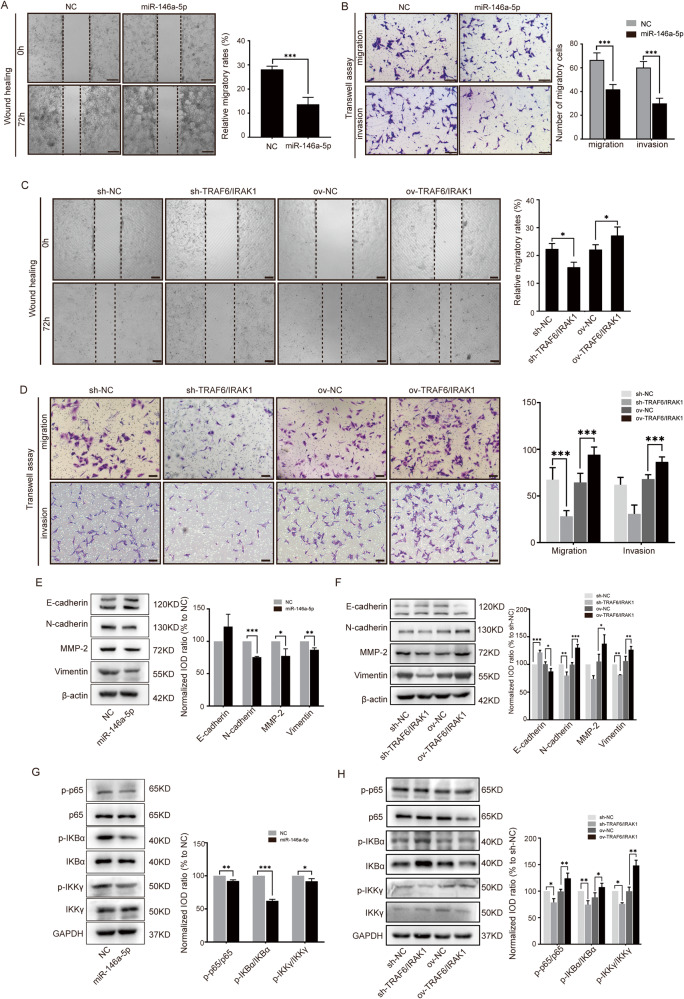
The miR-146a-5p regulated TRAF6-IRAK1 complex and the IKK-dependent NF-κB pathway. Wound healing (**A**, **C**) and transwell assays (**B**, **D**) were used to estimate the invasive and migratory abilities of U87MG^miR-146a-5p^, U87MG^ov-TRAF6/IRAK1^, and U87MG^sh-TRAF6/IRAK1^ cells compared with the corresponding controls. Scale bar = 200 μm. **E**, **F** Western blotting analysis as used to evaluate the levels of E-cadherin, N-cadherin, MMP-2, and vimentin in U87MG^miR-146a-5p^, U87MG^ov-TRAF6/IRAK1^, and U87MG^sh-TRAF6/IRAK1^ cells and their corresponding controls. **G**, **H** Western blotting was conducted to measure the expression of IKKγ, phosphorylated-IKKγ (p-IKKγ), IKBα, p-IKBα, p65, and p-p65 in U87MG^miR-146a-5p^, U87MG^ov-TRAF6/IRAK1^, and U87MG^sh-TRAF6/IRAK1^ cells and their corresponding controls. The ratios of p-IKKγ/IKKγ, p-IKBα/IKBα, and p-p65/p65 were calculated. *n* = 3, *p < 0.05, **p < 0.01, ***p < 0.001.

The EMT behaviors of U87MG^miR-146a-5p^ cells, U87MG^sh-TRAF6/IRAK1^ and U87MG^ov-TRAF6/IRAK1^ cells were estimated with Transwell and wound healing assays. MiR-146a-5p mitigated the migratory and invasive abilities of U87MG cells (Fig. 6A, B). For its targets, the EMT behaviors of U87MG^sh-TRAF6/IRAK1^ cells also mitigated while U87MG^ov-TRAF6/IRAK1^ cells exhibited enhanced migration and invasion abilities (Fig. 6C, D). The EMT marker expressions in these cells with miR-146a-5p overexpression or TRAF6/IRAK1 knockdown were markedly inhibited and increased in cells with TRAF6/IRAK1 overexpression (Fig. 6E, F). IRAK1 and TRAF6 are important components in NF-κB signaling pathway activation [21]. According to the analysis in inBio Discover, TRAF6 and IRAK1 are closely related to IKK complex recruitment (Supplementary Fig. S6). The NF-κB signaling pathway is regarded as downstream and it has been reported that IKKγ, the regulatory subunit of the IKK complex, is the substrate of E3 ubiquitin ligase. Therefore, the IKKγ-dependent NF-κB pathway was verified in U87MG^sh-TRAF6/IRAK1^ and U87MG^ov-TRAF6/IRAK1^ cells by Western blotting. When TRAF6 and IRAK1 were simultaneously knocked down, the phosphorylated IKKγ (p-IKKγ)/IKKγ, p-IKBα/IKBα, and p-p65/p65 ratios were decreased. In contrast, when TRAF6 and IRAK1 were simultaneously overexpressed, the p-IKKγ/IKKγ, p-IKBα/IKBα, and p-p65/p65 ratios were increased. Meanwhile, when miR-146a-5p was overexpressed, the same changes were observed in the NF-κB signaling pathway as in cells with sh-TRAF6/IRAK1 (Fig. 6G, H). The above findings suggested that miR-146a-5p regulated EMT in GBM cells by binding to IRAK1 and TRAF6 and the downstream IKKγ-dependent NF-κB signaling pathway. Deficiency of miR-146a-5p in M2-EVs disinhibited the NF-κB promoting EMT behaviors of GBM cells.

### miR-146a-5p and TRAF6-IRAK1 Expressions influenced the progression of GBM

**Fig. 7:**
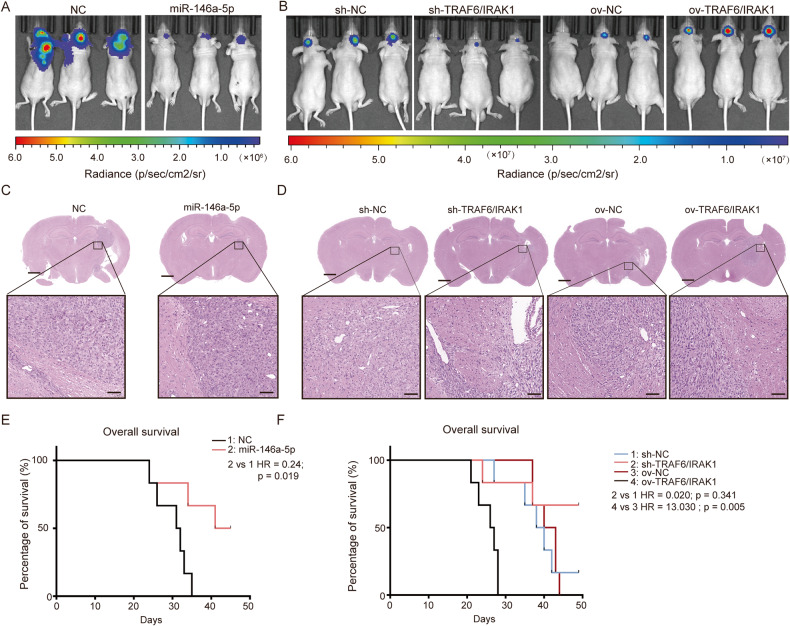
The miR-146a-5p and TRAF6-IRAK1 complex influence GBM progression in vivo. Nude mice bearing orthotopic U87MG-Luc GBM homografts were employed to evaluate the effect of miR-146a-5p and TRAF6-IRAK1 complex on the progression of GBM. Nude mouse brains were implanted with U87MG-Luc^NC^, U87MG-Luc^miR-146a-5p^, U87MG-Luc^ov-NC^, U87MG-Luc^sh-NC^, U87MG-Luc^ov-TRAF6/IRAK1^, and U87MG-Luc^sh-TRAF6/IRAK1^ cells Live animal images were acquired 3 weeks after GBM cell implantation. The luminescence intensities represented the tumor sizes and were used to evaluate the progression of GBM in vivo. **A** shows the comparison between U87MG-Luc^NC^ and U87MG-Luc^miR-146a-5p^. **B** shows the comparison among U87MG-Luc^ov-NC^, U87MG-Luc^sh-NC^, U87MG-Luc^ov-TRAF6/IRAK1^, and U87MG-Luc^sh-TRAF6/IRAK1^ cells. **C**, **D** After the mice were sacrificed, brain samples were collected, and H&E staining was performed to inspect the histological features of GBM tumors. The upper panel shows the whole fields of brain slices (scale bar = 1 mm) and the lower panel shows the magnified fields (scale bar = 50 μm). **E**, **F** The survival curves of tumor-bearing mice were generated based on the survival time of individual mice.

To validate the influence of TRAF6 and IRAK1 on GBM progression in vivo, an allogeneic U87MG-Luc GBM mouse model was employed. To simulate the impact of miR-146a-5p, U87MG-Luc^NC^, U87MG-Luc^miR-146a-5p^, U87MG-Luc^sh-NC^, U87MG-Luc^ov-NC^, U87MG-Luc^sh-TRAF6/IRAK1^, and U87MG-Luc^ov-TRAF6/IRAK1^ cells were implanted into nude mice in situ. Three weeks after implantation, tumors were detected in vivo with a small animal live imaging system. Compared with control GBM tumors, the intensities of mice with U87MG-Luc^miR-146a-5p^ GBM cells were significantly weaker than those of mice with U87MG-Luc^NC^. U87MG-Luc^sh-TRAF6/IRAK1^ GBM tumors were smaller with weaker luminescence intensities, and U87MG-Luc^ov-TRAF6/IRAK1^ tumors were larger and had higher luminescence intensities (Fig. 7A, B). H&E staining showed that U87MG-Luc^miR-146a-5p^ and U87MG-Luc^sh-TRAF6/IRAK1^ tumors had clear boundaries and surrounding normal tissue was suppressed, while U87MG-Luc^ov-TRAF6/IRAK1^ tumors infiltrated into normal tissue with a blurred boundary (Fig. 7C, D). Moreover, the survival of mice bearing these different GBM tumors differed significantly (Fig. 7E, F). All mice bearing U87MG-Luc^miR-146a-5p^ tumors were alive after ~6 weeks. No mice bearing U87MG-Luc^ov-TRAF6/IRAK1^ GBM tumors were alive after approximately 4 weeks. More than half of the mice bearing U87MG-Luc^sh-TRAF6/IRAK1^ GBM tumors were alive while only one mouse bearing U87MG-Luc^sh-NC^ GBM tumors was alive at the end of the experiment (7 weeks). The HR of ov-TRAF6/IRAK1 was 13.03, illustrating that the deficiency of miR-146a-5p and high expression of TRAF6-IRAK1 are important risk factors for GBM progression.

## Discussion

This work demonstrated that M2-EVs promoted GBM progression due to miR-146a-5p deficiency, which was elevated in M1-EVs. MiR-146a-5p deficiency in GAM-EVs reduced the invasion and migration of GBM cells through disinhibition of the TRAF6-IRAK1 complex and its downstream IKK-γ dependent NF-κB signaling pathway. In this study, we obtained a better understanding of GAMs with different immunological characteristics on the malignant progression of GBM.

In the past, the effect of GAMs on GBM has been widely studied. However, the complicated milieu and its interactions remain poorly understood. Single-cell RNA (scRNA) sequencing revealed that macrophages are the most common immune cell type in GBM [6, 22]. Due to the significant heterogeneity of GAMs, which are educated by the TIME [23, 24], it is difficult to precisely determine the activation states and transcriptional signatures of GAMs. Analysis of clinical specimens showed that GBMs contained a mixture of M1/M2-like GAMs [25]. We examined the mRNA levels of typical immunostimulatory and immunosuppressive gene markers from public databases and clinical samples and showed that immunosuppressive macrophage-related genes were upregulated in GBM compared with LGG and that the immunosuppressive gene sets significantly influenced the survival time of GBM patients, which was consistent with previous studies [26]. Gene coexpression network analysis based on single-cell profiles confirmed that GAMs, especially those with the M2-like phenotype, are negatively associated with glioma survival [27]. Accordingly, it is necessary to evaluate the function of GAMs during the progression of GBM based on their immunological features. The immunosuppressive characteristics of GAMs are the predominant results of education by the TIME of GBM [28]. The establishment of M1- and M2-like GAMs in vitro helped analyze the differences in GAMs and explore key targets under these opposite immune environments during our study.

EVs derived from GAMs are one of the most important vehicles for communication between GAMs and GBM cells. Recently, the effect of GBM-related EVs and their cargos has attracted increasing attention in terms of diagnosis [29], the exploration of underlying mechanisms [30], and the exploitation of new therapies [31]. However, studies focused on GAM-EVs are still in the initial stage, although the role of tumor modulators of GAMs is widely accepted [26]. Since it is challenging to isolate EVs derived from specific GAMs from clinical samples, studies lack data helpful for mapping the overall landscape of GAM-EVs in vivo, which hinders progression in the exploration of GAM-EVs. Furthermore, the mechanism by which cargos are loaded into EVs remains unclear. Specifically, increasing the expression of specific miRNAs and proteins in parent cells may not increase their contents in EVs, which limits the modulation of EVs in vivo, although the development of engineered exosomes has progressed dramatically [32]. By seeking to identify the main cargos in EVs, we hope that these cargos can be targeted directly in future studies.

Various miRNAs in EVs are effectors that regulate the biological behaviors of GBM. Research has shown that a series of miRNAs enhanced the transition of glioma stem cells from proneural to mesenchymal [33]; that miR-21 facilitates the invasion, proliferation, and migration of glioma cells [34]; and that miR-92a and miR-15a in M2-EVs impaired the abilities of invasion and migration in GBM cells [35]. In our study, to identify valuable miRNAs, we compared the differences in the types and quantities of miRNAs derived from M1-EVs and M2-EVs. MiR-146a-5p is considered an adaptive immunity-related miRNA. The human MIR146A gene, which is highly upregulated when exposed to IL-1β, LPS, and TNF-α, is observed to be NF-κB responsive in cells [36]. Healthy aging and inflammation are improved by miR-146a via its influence on canonical and noncanonical NF-κB pathways [37]. It critically impacts oncogenesis and tumor progression in various cancers [38]. MiR-146a-5p is regarded as an antitumor molecule in numerous cancers, such as esophageal cancer and CML, but is considered an oncomiR in bladder and cervical cancers, as well as melanoma. Previous research has confirmed that overexpression of miR-146a-5p in glioma cells led to inhibited growth [39] and enhanced apoptosis [40]. These results showed that the progression of GBM can be limited by miR-146a-5p, similar to our results. Furthermore, we revealed that immunostimulatory GAMs were a vital source for miR-146a-5p and that its level was notably lower in M2-EVs than in M1-EVs. MiR-146a-5p deficiency in both GBM cells and TIME is one of the deterministic factors that promotes GBM progression.

In this work, miR-146a-5p derived from GAM-EVs inhibited the EMT behaviors of GBM cells by inhibiting the activation of the TRAF6-IRAK1 complex. IRAK1 and TRAF6 are classical miR-146a target genes, although their relationship seemed to be conditional [41]. As we found in this work, miR-146a-5p influenced the expression of TRAF6 and IRAK1 at both mRNA and protein levels in vitro, although the analyzation on silicon is inconsistency. But the interaction between TRAF6 and IRAK1 was complicated. In both LGG and GBM, according to the analysis of public transcriptome databases, the TRAF6 and IRAK1 expression levels were positively correlated. And in the GBM protein database, TRAF6 and IRAK1 were not remarkably correlated. Interestingly, we established TRAF6 or IRAK1 knockdown and overexpression cell lines and evaluated the mRNA expression of these genes and found that the changes in TRAF6 and IRAK1 expression were not noticeably reciprocally influenced, while the changes were significant at the protein level. TRAF6 is an E3-ubiquitin ligase, and IRAK1 is regarded as the substrate for TRAF6 [21, 42]. TRAF6 is able to catalyze the formation of polyubiquitin linked at both Lys63 and Lys48 [42] in IRAK1. Lys48-linked ubiquitination facilitates subsequent proteasomal degradation in the ubiquitin-proteasome pathway while TRAF6-induced Lys63 ubiquitination is necessary for IRAK1-induced activation of the NF-κB signaling pathway [43]. Whereas, during this interaction, TRAF6 is also conditional downregulated through a proteasome-dependent pathway that is facilitated by IRAK1, which depends on the distance between the N-terminal death domain and the TRAF6 -binding site in IRAK1 [44, 45]. Meanwhile, IRAK1 promotes oligomerization of TRAF6. It is regarded as a *brake* of excessive activation of the NF-κB pathway. During our observation, the expression pattern of TRAF6 and IRAK1 in GBM samples were not banded together. Both of them have specific spatial expression characteristics, which explains why there was no significant correlation between TRAF6 and IRAK1 at the protein level in GBM tissue. Compared with the leading edge of GBM, cellular tumors expressed more TRAF6 and IRAK1 enhanced the activation of the NF-κB signaling pathway and then increased the invasive ability of GBM cells.

At the transcriptome level, bulk mRNA sequencing data hardly reflect the features led by high heterogeneity, although we noticed that the mRNAs of TRAF6 and IRAK1 were positively correlated. Only one GBM case in Ivy GAP had high miR-146a levels, and the expression levels of TRAF6 and IRAK1 were relatively lower. We observed that miR-146a-5p binding diminished the expression of both IRAK1 and TRAF6 in vitro. All of the above results illustrate that miR-146a-5p is capable of regulating the TRAF6-IRAK1 complex and its downstream NF-κB signaling pathway.

We suggest that miR-146a-5p is in a one-to-multi state with its binding genes, although previous studies hypothesized that the relationships between miRNAs and their target genes are one-to-one or multi-to-one. The deterministic factor of this modification is the quantity of miRNA according to our conjecture, which needs further validation. When TRAF6 and IRAK1 were knocked down simultaneously, NF-κB signaling pathway activation and EMT marker expression were significantly inhibited. Compared with single-gene knockdown, dual-gene silencing more reliably simulated EVs miR-146a-5p biological function, and marked phosphorylation of the IKK-γ and NF-κB p65 subunits was observed.

Recently, M1-EVs have been considered carriers in GBM therapies, although a specific cargo was not mentioned in this study [46]. Repolarizing M2-like GAMs into M1-like GAMs with effective biomaterials (such as a virus-mimicking membrane-coated nucleic acid nanogel Vir-Gel [47] and bovine serum albumin-MnO2-SAL [48]) or active molecules (such as Galectin-9 siRNA [49] and curcumin [50]) that were able to be transferred into the TIME and to exert therapeutic functions were considered a promising approach for GBM treatment. Our findings demonstrated that elevating miR-146a-5p levels in the TIME might be a novel therapeutic approach for GBM.

## Materials and methods

### Collection of public glioma data and online analysis

Expressions, correlations, and patients’ overall survival (OS) of interested genes in TCGA databases were analyzed with GEPIA 2.0 (http://gepia2.cancer-pku.cn/#index). The interactions of proteins were analyzed with STRING 11.5 (https://cn.string-db.org/) and inBio Discover (https://inbio-discover.com/). The spatial features of GBM transcriptomes were analyzed with Ivy GAP (http://glioblastoma.alleninstitute.org/). The miRNA and mRNA data of GBM and LGG patients in the CGGA were analyzed with an online tool (http://www.cgga.org.cn/analyse/) and Sanger Box (http://sangerbox.com/home.html). CPTAC.

### Patient samples

Samples were collected from twelve patients who had surgically resected gliomas between 2013 and 2023 at Chongqing General Hospital and were histologically confirmed postoperatively as glioma. The assigned glioma grades were based on pathological diagnosis under the WHO Classification of Tumors, 5th Edition, Volume 6: Tumors [51]. The Ethics Committee of Chongqing General Hospital supervised and approved this study in compliance with the Declaration of Helsinki. Patients (or their representatives) who were involved signed the individual informed consent form.

### Cell lines and reagents

U87MG and A172 cells, the human GBM cell lines, were cultured with DMEM (Gibco, Carlsbad, USA) with 10% fetal bovine serum (FBS; Gibco), penicillin 50 units/ml, and streptomycin 50 μg/ml (Beyotime Biotechnology, Shanghai, China). U87MG-Luc cell lines were established with pLX313-Firefly, a luciferase plasmid (Addgene #118017, Watertown, USA). The human monocyte cell line THP-1 was cultured in RPMI 1640 (Gibco) with 10% FBS (Gibco), penicillin 50 units/ml, and streptomycin 50 μg/ml. THP-1 cells were pretreated with phorbol myristate acetate (PMA; Sigma‒Aldrich, St. Louis, USA) for 48 h to obtain activated macrophage cells. GAM polarization was conducted in accordance with previously reported procedures with some modifications [52]. In brief, to obtain M1-GAMs, PMA-stimulated THP-1 cells were cultured with granulocyte-macrophage colony-stimulating factor (GM-CSF; PeproTech, Cranbury, USA) at 50 ng/mL for three days. IL-4 and IL-13 (PeproTech) were used to stimulate the generation of M2-GAMs. Lipo 2000 (GlpBio, Montclair, USA) was used to transfect the inhibitor negative control sequence (NC), has-miR-146a-5p inhibitor, mimic NC, and has-miR-146a-5p mimic into U87MG and A172 cells. The sequences are listed in Supplementary Table S2.

### EV extraction

EVs were extracted from the FBS-free conditioned medium of M1-GAMs and M2-GAMs and extracted by ultracentrifugation with an Optima MAX-XP instrument (Beckman, Indianapolis, USA) as previously described [53, 54]. In brief, the cell culture supernatant was first centrifuged at 300×*g* for 10 min to remove whole cells, and then a 2000×*g* centrifuge for 10 min was performed to remove dead cells. To remove cell debris, the supernatant was ultracentrifuged for 30 min at 10,000×*g* and ultracentrifuged for 70 min at 100,00×*g* to isolate EVs. Finally, the pellet was washed with phosphate-buffered saline (PBS). Retained EVs were then analyzed and used for follow-up studies.

### Transmission electron microscopy and nanoparticle tracking analysis

Transmission electron microscopy (TEM) was used to observe the morphological features of EVs that were negatively stained. After being fixed with 1% glutaraldehyde and washed, EVs were placed on Formvar/carbon-coated 300-mesh copper grids and incubated at room temperature for 1 min. Uranyl acetate stained EVs for 1 min. Images were acquired by TEM (JEM-1400Plus, JEOL, Tokyo, Japan) after the grids were washed and dried.

The concentration of EVs and their size distribution were estimated by NTA (NanoSight NS300, Malvern Panalytical, Malvern, UK). The detailed procedure followed the manufacturer’s instructions, as previously described [55]. Data were analyzed with Zetasizer software (Malvern Instruments).

### EV internalization

To confirm that M1-EVs and M2-EVs were internalized by GBM cells, EVs were stained and labeled with Dil (Beyotime Biotechnology) following the manual and visualized with an LSM880 laser confocal microscope (Zeiss, Cambridge, UK). They were ultracentrifuged at 100,000×g for 60 min to remove residual dye and resuspended. Then, U87MG cells were cocultured with Dil-labeled EVs for 12 h. After refreshing the culture medium, Alexa Fluor™ 488 phalloidin (Invitrogen, Waltham, USA) and Hoechst (Beyotime Biotechnology) were added to label cells and EV internalization was observed after washing.

### MiRNA sequencing of EVs

Isolated M1-EVs and M2-EVs were subjected to miRNA-Seq analysis (GCBI, Shanghai, China). The differential expression of miRNAs was analyzed with DEGseq and uploaded to the NCBI Gene Expression Omnibus (GEO) database (GSE212171, www.ncbi.nlm.nih.gov/geo). Heatmaps showing the differentially regulated miRNAs were generated using online tools provided by GCBI (https://biosys.bgi.com/), and a volcanic plot was developed to visualize the significance and change fold of EV miRNAs.

### Prediction of the miR-146a-5p binding genes

Six online miRNAs databases, namely, miRWalk (http://mirwalk.umm.uni-heidelberg.de/), miRDB (http:// mirdb.org), TargetScan 8.0 (https://www.targetscan.org/vert_80/), RNA22 (https://cm.jefferson.edu/rna22/), ENCORI (https://starbase.sysu.edu.cn/), and miRTarBase (https://mirtarbase.cuhk.edu.cn/~miRTarBase/miRTarBase_2022/php/index.php), predicted the miR-146a-5p binding genes and the predictions were merged.

### Dual-luciferase reporter assay

Two fragments of the wild-type (WT) TRAF6 3′ untranslated region (UTR), one fragment of the WT IRAK1 3′UTR, and the corresponding MT sequences were used to construct the WT and mutant (MT) pmir-GLO luciferase vectors. The plasmids containing the WT TRAF6 or IRAK1 3′UTR or the corresponding 3′UTRs with miRNA binding site mutations driven by the pmirGLO luciferase reporter and the mimic NC and has-miR-146a-5p mimic were cotransfected in U87MG cells with Lipo 2000. After 24 hours, the relative luciferase activity was detected by a Dual-Luciferase Reporter Assay Kit (Promega, Madison, USA) and a Varioskan Flash microplate reader (Thermo Scientific, Waltham, USA) following the manufacturer’s instructions.

### TRAF6 and IRAK1 knockdown and overexpression, and has-miR-146a-5p overexpression

ShRNA plasmids were used to interfere with the translation of TRAF6 and IRAK1. The TRAF6 and IRAK1 shRNA sequences were inserted into pLVX-shRNA2 (Clontech 632179, Mountain View, USA). The vector pLVX-CMV-IRES-PURO was employed to construct the TRAF6 and IRAK1 overexpression plasmids. (The primer and shRNA sequences are listed in Supplementary Table S4). The vector GV298 (U6-MCS-Ubiquitin-Cherry-IRES-puromycin) was employed to construct the has-miR-146a-5p overexpression plasmid. All plasmids were transformed into *E. coli* strain Stbl3. The lentivirus was then packaged in HEK293T cells with psPAX2 (Addgene #12260) and pMD2.G (Addgene #12259). After transfection, HEK293T cells were incubated for 48 h. The supernatant with virus particles was collected. After being filtered, the virus particles were added to infect U87MG and A172 cells for infection. Successfully infected cells were screened with puromycin. To validate the knockdown or overexpression of IRAK1 and TRAF6, Western blot assays were conducted.

### Quantitative real-time polymerase chain reaction

TRIzol (Invitrogen, USA) was used to prepare the total RNA of cells. An RNAprep Pure Cell Kit (Tiangen Biotech, Beijing, China) and Super M-MuLV First Strand cDNA Synthesis Mix (Sangon Biotech, Shanghai, China) were used for mRNA isolation. MiRNA was prepared with a SanPrep Column microRNA Extraction Kit and a microRNA First Strand cDNA Synthesis Kit (Sangon Biotech). 2X SYBR Abstract PCR Mix and MicroRNAs qPCR Kit (Sangong Biotech) were used for RT-PCR. The thermal cycling protocol used for quantitative RT‒PCR was as follows: predenaturation at 94 °C for 5 min, 40 cycles of denaturation at 94 °C for 30 s, annealing at 57 °C for 30 s, and extension at 72 °C for 30 s. The primer sequences are listed in Supplementary Table S3.

### Western blot assays

Prechilled RIPA buffer (Beyotime Biotechnology) was used to prepare total protein. Extracted protein was separated by SDS‒PAGE, and transferred to polyvinylidene fluoride (PVDF) membranes. The membranes were blocked with 5% nonfat milk for 2 h at room temperature and incubated with specific primary antibodies (information regarding the primary antibodies is listed in Supplementary Table S5) at 4 °C overnight. Then, corresponding HRP-conjugated secondary antibodies (Beyotime Biotechnology) were used in the membrane incubation at room temperature for 1 h. Enhanced chemiluminescence was used for visualization. The original data are available in the Supplementary Material.

### Wound healing assay and Transwell assay

Wound healing assays were applied to estimate GBM cell migratory ability. U87MG and A172 cells were seeded and cultured in six-well plates until 100% confluence. These cells were cultured with FBS-free medium for 24 h before the experiment. The wounds were scratched with 200 μL pipette tips, and then the cells were washed 3 times and cultured with FBS-free medium. Wound healing was observed at 72 h with an optical microscope (IX71, Olympus, Tokyo, Japan). ImageJ software was used to estimate and quantify the healing rates.

Transwell assays were used to estimate GBM cell migratory and invasive abilities with chambers that have 0.4 μm micropores (Corning Life Sciences, NY, USA). For invasion assays, U87MG and A172 cells were seeded in the upper chambers of each insert, which contained a Matrigel-coated membrane (Corning Life Sciences), and cultured with FBS-free medium. Medium containing 10% FBS was added to the lower chambers of the 24-well plates. For migration assays, GBM cells were seeded in the upper chambers without Matrigel coating on the membrane. After incubation for 15 h at 37 °C and fixation in paraformaldehyde, these cells were stained with crystal violet solution. Afterward, the number of cells on the lower surface of each membrane was determined and averaged in 5 random fields.

### Bimolecular fluorescence complementation assay

A BiFC assay was conducted to verify that TRAF6 and IRAK1 interacted with each other. TRAF6 and IRAK1 cDNA were inserted into the vectors pBiFC-VN173 (Addgene #22010) and pBiFC-CC155 (Addgene #22015) to construct the plasmids. HEK293T cells were transfected for 24 h with pBiFC-VN173-TRAF6, pBiFC-VN173-IRAK1, pBiFC-CC155-TRAF6, and pBiFC-CC155-IRAK1 alone; cotransfected with pBiFC-VN173-TRAF6 and pBiFC-CC155-IRAK1; or cotransfected with pBiFC-VN173-IRAK1 and pBiFC-CC155-TRAF6. The fluorescence intensity was then determined with a laser-scanning fluorescence microscope.

### Coimmunoprecipitation

Total protein was prepared with Co‐IP lysis buffer (Beyotime Biotechnology). Equal amounts of total protein from each sample were incubated with primary antibodies (information regarding the primary antibodies is listed in Supplementary Table S5) at 4 °C overnight. Then, Protein A/G PLUS-Agarose (Santa Cruz Biotechnology, Dallas, USA) was added and incubated with shaking at 4 °C for 3 h. The bound proteins were eluted with SDS-loading buffer after washing. Immunoprecipitated proteins were used in immunoblotting following procedures described in the Western blotting section above. (Information regarding the primary antibodies is listed in Supplementary Table S5).

### Orthotopic homograft mouse model

The animal experimental procedures were supervised and approved by the ethics committee of Chongqing General Hospital. Male BALB/c-nude mice (4–5 weeks) were used in the research. Forty-four mice were randomly divided into four groups, and 5 μL of U87MG-Luc^NC^, U87MG-Luc^ov-miR-146a-5p^, U87MG-Luc^sh-NC^, U87MG-Luc^ov-NC^ U87MG-Luc^ov-TRAF6/IRAK1^, or U87MG-Luc^sh-TRAF6/IRAK1^ cells (1 × 10^5^) was injected in situ into the brain by using a small animal stereotactic injector (RWD, Shenzhen, China). Mice were fed for 3 weeks, and the tumor sizes and luminescence intensity were measured with a small animal live imaging system (IVIS® Spectrum CT, PerkinElmer, USA). The survival times of mice bearing GBM tumors were recorded, and the tumors were harvested for detection of tumor invasion and analysis of protein expression.

### Immunohistochemical, immunofluorescence, and H&E staining

GBM specimens obtained from patients were used to detect CD163, CD206, TRAF6, and IRAK1 using IHC staining. The primary steps were as follows: fixation with paraformaldehyde, embedding in paraffin, dewaxing in xylene, rinsing in graded ethanol solutions and rehydration. Next, antigen retrieval was performed and the slides were pretreated with sodium citrate buffer at 95 °C for 15 min. After washing with PBS 3 times, the slides were stained with primary antibodies against CD163 and MSR1 (information regarding the primary antibodies is listed in Supplementary Table S5) at 4 °C overnight. After being washed again, the slides were immersed in buffer containing HRP-conjugated anti-mouse/rabbit antibodies for 2 h. DAB chromogen solution was added and then incubated for approximately 1 min. The numbers of CD163- and MSR1-positive cells in 5 random fields were determined and analyzed in images on the same magnification scale.

Frozen GBM slides obtained from patients were used to detect TRAF6 and IRAK1 using IF staining. The slides were fixed with paraformaldehyde, and permeated with Tween-20. After washing with PBS 3 times, the slides were blocked with goat serum and stained with primary antibodies against TRAF6 and IRAK1 (information regarding the primary antibodies is listed in Supplementary Table S5) at 4 °C overnight. After being washed again, the slides were stained with CoralLite488-conjugated anti-mouse antibody or CoralLite594-conjugated anti-rabbit antibody for 2 h at room temperature. DAPI was used to stained nucleus for 10 min.

GBM slides derived from patients and brain samples harvested from mice bearing GBM tumors were processed into paraffin sections and stained with H&E to observe the morphological features. For H&E staining, deparaffinization was completed with dimethylbenzene, followed by 3 min of hematoxylin and eosin staining. Then, the stained slices were dehydrated and visualized.

### Statistical analysis

All in vitro experiments were performed 3 times independently. IBM SPSS software 24.0 was used for the statistical analysis. Means and standard deviations were used to represent data (means ± SD). Comparisons between two groups were calculated by Student’s t test and comparisons among multiple groups were analyzed by ANOVA. The hazards ratio (HR) was calculated with Cox regression. P values less than 0.05 were regarded as statistically significant. The correlation analysis was performed with online tool Sangerbox (http://sangerbox.com/).

## Supplementary information

Supplementary_Table S1

Supplementary_Table S2

Supplementary_Table S3

Supplementary_Table S4

Supplementary_Table S5

Supplementary_Table S6

Supplementary Figures and legends

## Data Availability

The datasets generated during and/or analysed during the current study are available from the corresponding author on reasonable request.

## References

[1] Ostrom QT, Gittleman H, Truitt G, Boscia A, Kruchko C, Barnholtz-Sloan JS (2018). CBTRUS Statistical Report: primary brain and other central nervous system tumors diagnosed in the United States in 2011–2015. Neuro Oncol..

[2] Karachi A, Dastmalchi F, Mitchell DA, Rahman M (2018). Temozolomide for immunomodulation in the treatment of glioblastoma. Neuro Oncol.

[3] Tan AC, Ashley DM, López GY, Malinzak M, Friedman HS, Khasraw M (2020). Management of glioblastoma: state of the art and future directions. CA Cancer J Clin..

[4] Jackson CM, Choi J, Lim M (2019). Mechanisms of immunotherapy resistance: lessons from glioblastoma. Nat Immunol..

[5] Lathia JD, Mack SC, Mulkearns-Hubert EE, Valentim CL, Rich JN (2015). Cancer stem cells in glioblastoma. Genes Dev..

[6] DeCordova S, Shastri A, Tsolaki AG, Yasmin H, Klein L, Singh SK (2020). Molecular heterogeneity and immunosuppressive microenvironment in glioblastoma. Front Immunol..

[7] Gieryng A, Pszczolkowska D, Walentynowicz KA, Rajan WD, Kaminska B (2017). Immune microenvironment of gliomas. Lab Invest..

[8] Dunn GP, Old LJ, Schreiber RD (2004). The three Es of cancer immunoediting. Annu Rev Immunol..

[9] Chen Z, Feng X, Herting CJ, Garcia VA, Nie K, Pong WW (2017). Cellular and molecular identity of tumor-associated macrophages in glioblastoma. Cancer Res..

[10] Xuan W, Lesniak MS, James CD, Heimberger AB, Chen P (2021). Context-dependent glioblastoma-macrophage/microglia symbiosis and associated mechanisms. Trends Immuno.l.

[11] Pathria P, Louis TL, Varner JA (2019). Targeting tumor-associated macrophages in cancer. Trends Immunol..

[12] Gutmann DH, Kettenmann H (2019). Microglia/brain macrophages as central drivers of brain tumor pathobiology. Neuron..

[13] Hambardzumyan D, Gutmann DH, Kettenmann H (2016). The role of microglia and macrophages in glioma maintenance and progression. Nat Neurosci..

[14] Han C, Zhang C, Wang H, Zhao L (2021). Exosome-mediated communication between tumor cells and tumor-associated macrophages: implications for tumor microenvironment. Oncoimmunology..

[15] Wortzel I, Dror S, Kenific CM, Lyden D (2019). Exosome-mediated metastasis: communication from a distance. Dev Cell..

[16] Rooj AK, Mineo M, Godlewski J (2016). MicroRNA and extracellular vesicles in glioblastoma: small but powerful. Brain Tumor Pathol..

[17] Mondal A, Kumari Singh D, Panda S, Shiras A (2017). Extracellular vesicles as modulators of tumor microenvironment and disease progression in glioma. Front Oncol..

[18] Broekman ML, Maas SLN, Abels ER, Mempel TR, Krichevsky AM, Breakefield XO (2018). Multidimensional communication in the microenvirons of glioblastoma. Nat Rev Neurol..

[19] Cocks A, Del Vecchio F, Martinez-Rodriguez V, Schukking M, Fabbri M (2022). Pro-tumoral functions of tumor-associated macrophage EV-miRNA. Semin Cancer Biol..

[20] Choi KC, Lee YS, Lim S, Choi HK, Lee CH, Lee EK (2006). Smad6 negatively regulates interleukin 1-receptor-Toll-like receptor signaling through direct interaction with the adaptor Pellino-1. Nat Immunol..

[21] Conze DB, Wu CJ, Thomas JA, Landstrom A, Ashwell JD (2008). Lys63-linked polyubiquitination of IRAK-1 is required for interleukin-1 receptor- and toll-like receptor-mediated NF-kappaB activation. Mol Cell Biol..

[22] Cui X, Wang Q, Zhou J, Wang Y, Xu C, Tong F (2021). Single-cell transcriptomics of glioblastoma reveals a unique tumor microenvironment and potential immunotherapeutic target against tumor-associated macrophage. Front Oncol..

[23] Fu W, Wang W, Li H, Jiao Y, Huo R, Yan Z (2020). Single-cell atlas reveals complexity of the immunosuppressive microenvironment of initial and recurrent glioblastoma. Front Immunol..

[24] Cheng W, Ren X, Zhang C, Cai J, Liu Y, Han S (2016). Bioinformatic profiling identifies an immune-related risk signature for glioblastoma. Neurology..

[25] Zeiner PS, Preusse C, Golebiewska A, Zinke J, Iriondo A, Muller A (2019). Distribution and prognostic impact of microglia/macrophage subpopulations in gliomas. Brain Pathol..

[26] Baig MS, Roy A, Rajpoot S, Liu D, Savai R, Banerjee S (2020). Tumor-derived exosomes in the regulation of macrophage polarization. Inflamm Res..

[27] Zhang H, Luo YB, Wu W, Zhang L, Wang Z, Dai Z (2021). The molecular feature of macrophages in tumor immune microenvironment of glioma patients. Comput Struct Biotechnol J..

[28] Pyonteck SM, Akkari L, Schuhmacher AJ, Bowman RL, Sevenich L, Quail DF (2013). CSF-1R inhibition alters macrophage polarization and blocks glioma progression. Nat Med..

[29] Müller Bark J, Kulasinghe A, Chua B, Day BW, Punyadeera C (2020). Circulating biomarkers in patients with glioblastoma. Br J Cancer..

[30] Himes BT, Peterson TE, de Mooij T, Garcia LMC, Jung MY, Uhm S (2020). The role of extracellular vesicles and PD-L1 in glioblastoma-mediated immunosuppressive monocyte induction. Neuro Oncol..

[31] Basu B, Ghosh MK (2019). Extracellular vesicles in glioma: from diagnosis to therapy. Bioessays..

[32] Wu JY, Li YJ, Hu XB, Huang S, Luo S, Tang T (2021). Exosomes and biomimetic nanovesicles-mediated anti-glioblastoma therapy: a head-to-head comparison. J Control Release..

[33] Zhang Z, Xu J, Chen Z, Wang H, Xue H, Yang C (2020). Transfer of microRNA via macrophage-derived extracellular vesicles promotes proneural-to-mesenchymal transition in glioma stem cells. Cancer Immunol Res..

[34] Yang F, Wang T, Du P, Fan H, Dong X, Guo H (2020). M2 bone marrow-derived macrophage-derived exosomes shuffle microRNA-21 to accelerate immune escape of glioma by modulating PEG3. Cancer Cell Int..

[35] Yao J, Wang Z, Cheng Y, Ma C, Zhong Y, Xiao Y (2021). M2 macrophage-derived exosomal microRNAs inhibit cell migration and invasion in gliomas through PI3K/AKT/mTOR signaling pathway. J Transl Med..

[36] Taganov KD, Boldin MP, Chang KJ, Baltimore D (2006). NF-kappaB-dependent induction of microRNA miR-146, an inhibitor targeted to signaling proteins of innate immune responses. Proc Natl Acad Sci Usa..

[37] Olivieri F, Prattichizzo F, Giuliani A, Matacchione G, Rippo MR, Sabbatinelli J (2021). miR-21 and miR-146a: the microRNAs of inflammaging and age-related diseases. Ageing Res Rev..

[38] Iacona JR, Lutz C (2019). S. miR-146a-5p: Expression, regulation, and functions in cancer. Wiley Interdiscip Rev Rna..

[39] Mei J, Bachoo R, Zhang CL (2011). MicroRNA-146a inhibits glioma development by targeting Notch1. Mol Cell Biol..

[40] Wu H, Liu Q, Cai T, Chen YD, Wang ZF (2015). Induction of microRNA-146a is involved in curcumin-mediated enhancement of temozolomide cytotoxicity against human glioblastoma. Mol Med Rep..

[41] Bertolet G, Kongchan N, Miller R, Patel RK, Jain A, Choi JM, et al. MiR-146a wild-type 3′ sequence identity is dispensable for proper innate immune function in vivo. Life Sci Alliance 2 (2019). 10.26508/lsa.201800249. 10.26508/lsa.201800249 PMC6379685 30777858

[42] Yang WL, Wang J, Chan CH, Lee SW, Campos AD, Lamothe B (2009). The E3 ligase TRAF6 regulates Akt ubiquitination and activation. Science.

[43] Muroi M, Tanamoto K (2008). TRAF6 distinctively mediates MyD88- and IRAK-1-induced activation of NF-kappaB. J Leukoc Biol..

[44] Muroi M, Tanamoto K (2012). IRAK-1-mediated negative regulation of Toll-like receptor signaling through proteasome-dependent downregulation of TRAF6. Biochim Biophys Acta..

[45] Watanabe S, Zenke K, Sugiura Y, Muroi M (2022). Minimal structure of IRAK-1 to induce degradation of TRAF6. Immunobiology..

[46] Wang X, Ding H, Li Z, Peng Y, Tan H, Wang C (2022). Exploration and functionalization of M1-macrophage extracellular vesicles for effective accumulation in glioblastoma and strong synergistic therapeutic effects. Signal Transduct Target Ther..

[47] Gao X, Li S, Ding F, Liu X, Wu Y, Li J (2021). A virus-mimicking nucleic acid nanogel reprograms microglia and macrophages for glioblastoma therapy. Adv Mater..

[48] Liang F, Zhu L, Wang C, Yang Y, He Z (2021). BSA-MnO(2)-SAL multifunctional nanoparticle-mediated M(1) macrophages polarization for glioblastoma therapy. RSC Adv..

[49] Li C, Guan N, Liu F (2023). T7 peptide-decorated exosome-based nanocarrier system for delivery of Galectin-9 siRNA to stimulate macrophage repolarization in glioblastoma. J Neurooncol..

[50] Mukherjee S, Fried A, Hussaini R, White R, Baidoo J, Yalamanchi S (2018). Phytosomal curcumin causes natural killer cell-dependent repolarization of glioblastoma (GBM) tumor-associated microglia/macrophages and elimination of GBM and GBM stem cells. J Exp Clin Cancer Res..

[51] Louis DN, Perry A, Wesseling P, Brat DJ, Cree IA, Figarella-Branger D (2021). The 2021 WHO classification of tumors of the central nervous system: a summary. Neuro Oncol..

[52] Lei X, Chen M, Li X, Huang M, Nie Q, Ma N (2018). A vascular disrupting agent overcomes tumor multidrug resistance by skewing macrophage polarity toward the M1 phenotype. Cancer Lett..

[53] Théry, C, Amigorena, S, Raposo, G & Clayton, A Isolation and characterization of exosomes from cell culture supernatants and biological fluids. Curr Protoc Cell Biol. 2006;Chapter 3:Unit 3.22. 10.1002/0471143030.cb0322s30. 10.1002/0471143030.cb0322s30 18228490

[54] Hoshino A, Costa-Silva B, Shen TL, Rodrigues G, Hashimoto A, Tesic Mark M (2015). Tumour exosome integrins determine organotropic metastasis. Nature..

[55] Zhang X, Sai B, Wang F, Wang L, Wang Y, Zheng L (2019). Hypoxic BMSC-derived exosomal miRNAs promote metastasis of lung cancer cells via STAT3-induced EMT. Mol Cancer..

