# Supplementary material for: MiR-146a-5p deficiency in extracellular vesicles of glioma-associated macrophages promotes epithelial-mesenchymal transition through the NF-κB signaling pathway
Source: Cell Death Discov. 2023 Jun 30;9:206. doi: 10.1038/s41420-023-01492-0 (PMC10313823; doi:10.1038/s41420-023-01492-0)
Supplement: Supplementary file 2 — Supplementary_Table S2 [file 41420_2023_1492_MOESM2_ESM.docx]

Additional files :Table S1 The sequences of the miR-146a-5p mimic and inhibitor

| Mimic NC | Sense(5'-3') | UUGUACUACACAAAAGUACUG |
| --- | --- | --- |
|  | Antisense(5'-3') | GUACUUUUGUGUAGUACAAUU |
| hsa-miR-146a-5p mimic | Sense(5'-3') | UGAGAACUGAAUUCCAUGGGUU |
|  | Antisense(5'-3') | CCCAUGGAAUUCAGUUCUCAUU |
| Inhibitor NC | Sense(5'-3') | CAGUACUUUUGUGUAGUACAA |
| hsa-miR-146a-5p inhibitor | Sense(5'-3') | AACCCAUGGAAUUCAGUUCUCA |
