# Supplementary material for: MiR-146a-5p deficiency in extracellular vesicles of glioma-associated macrophages promotes epithelial-mesenchymal transition through the NF-κB signaling pathway
Source: Cell Death Discov. 2023 Jun 30;9:206. doi: 10.1038/s41420-023-01492-0 (PMC10313823; doi:10.1038/s41420-023-01492-0)
Supplement: Supplementary file 3 — Supplementary_Table S3 [file 41420_2023_1492_MOESM3_ESM.docx]

Additional Files: Table S3: The sequences of primers used for RT-qPCR detection

| **No.** | **Name** | **Sequence (5' -> 3')** | | **Tm (℃)** |
| --- | --- | --- | --- | --- |
| 1 | TNF-a | Forward Primer | CTCCTCTCTGCCATCAAGAGC | 59.6 |
|  |  | Reverse Primer | AAAGTAGACCTGCCCAGACTCG | 59.6 |
| 2 | TGF-β1 | Forward Primer | GTACCTGAACCCGTGTTGCTCT | 62.7 |
|  |  | Reverse Primer | GAACCCGTTGATGTCCACTTGC | 61.6 |
| 3 | CCL-2 | Forward Primer | CATAGCAGCCACCTTCATTCCC | 62.1 |
|  |  | Reverse Primer | CTGCACTGAGATCTTCCTATTGGT | 61.5 |
| 4 | TLR-4 | Forward Primer | TGTGCTGAGTTTGAATATCACC | 55.4 |
|  |  | Reverse Primer | CTTGAGTAGATAACAAAGGCATC | 54.5 |
| 5 | β-actin | Forward Primer | ACCCGCCGCCAGCTCACC | 68.5 |
|  |  | Reverse Primer | GGGGGGCACGAAGGCTCATC | 68.3 |
| 6 | IRAK1 | Forward Primer | TGTGCTCAGAACGGCTTCTA | 56.8 |
|  |  | Reverse Primer | GGCTGTACCCAGAAGGATGT | 56.3 |
| 7 | TRAF6 | Forward Primer | CTACAGCCCCAATTCCATGC | 60.3 |
|  |  | Reverse Primer | CCAGAGTCGGGTATAACGCT | 57.0 |
| 8 | hsa-miR146a-5p | Forward Primer | CGCGTGAGAACTGAATTCCA | 59.4 |
