# Supplementary material for: MiR-146a-5p deficiency in extracellular vesicles of glioma-associated macrophages promotes epithelial-mesenchymal transition through the NF-κB signaling pathway
Source: Cell Death Discov. 2023 Jun 30;9:206. doi: 10.1038/s41420-023-01492-0 (PMC10313823; doi:10.1038/s41420-023-01492-0)
Supplement: Supplementary file 4 — Supplementary_Table S4 [file 41420_2023_1492_MOESM4_ESM.docx]

Table S2 The shRNA sequences of TRAF6 and IRAK1

| **Name** | **Target sequence** | **shRNA Sequence (5' -> 3')** | |
| --- | --- | --- | --- |
| TRAF6 | TTCATAGTTTGAGCGTTATAC | sense | gatccGTTCATAGTTTGAGCGTTATACCTCGAGGTATAACGCTCAAACTATGAATTTTTG |
|  |  | Anti-sense | AATTCAAAAATTCATAGTTTGAGCGTTATACCTCGAGGTATAACGCTCAAACTATGAACg |
| IRAK1 | GCCCGAAGAAAGTGATGAATT | sense | gatccGCCCGAAGAAAGTGATGAATTCTCGAGAATTCATCACTTTCTTCGGGCTTTTTG |
|  |  | Anti-sense | AATTCAAAAAGCCCGAAGAAAGTGATGAATTCTCGAGAATTCATCACTTTCTTCGGGCg |
