# Supplementary material for: MiR-146a-5p deficiency in extracellular vesicles of glioma-associated macrophages promotes epithelial-mesenchymal transition through the NF-κB signaling pathway
Source: Cell Death Discov. 2023 Jun 30;9:206. doi: 10.1038/s41420-023-01492-0 (PMC10313823; doi:10.1038/s41420-023-01492-0)
Supplement: Supplementary file 5 — Supplementary_Table S5 [file 41420_2023_1492_MOESM5_ESM.docx]

Additional files: Table S4: Primary antibodies used in western blotting, CoIP, and IHC

1. For western blotting:

| Antigens | Manufacturer | Catalogue numbers | Application |
| --- | --- | --- | --- |
| CD63 | abcam | ab134045 | 1:1000 |
| CD9 | abcam | ab223052 | 1:1000 |
| CD81 | abcam | ab109201 | 1:1000 |
| E-cadherin | Proteintech | 20874-1-AP | 1:5000 |
| N-cadherin | Proteintech | 22018-1-Ap | 1:5000 |
| MMP-2 | Cell Signaling Technology | 40994S | 1:1000 |
| Vimentin | Proteintech | 10366-1-AP | 1:2500 |
| IRAK1 | Proteintech | 10478-2-AP | 1:2500 |
| TRAF6 | Cell Signaling Technology | 8028S | 1:1000 |
| p65 | Cell Signaling Technology | 8242S | 1:1000 |
| p-p65 | Cell Signaling Technology | 3039S | 1:1000 |
| IKB-α | Cell Signaling Technology | 4814S | 1:1000 |
| p-IKB-α | Cell Signaling Technology | 9246S | 1:1000 |
| IKK-γ | Cell Signaling Technology | 2685S | 1:1000 |
| p-IKK-γ | Cell Signaling Technology | 2689S | 1:1000 |
| β-actin | Proteintech | 66009-1-lg | 1:2500 |
| GAPDH | Proteintech | 10494-1-AP | 1:2500 |

2. For IHC:

| CD163 | Servicebio | GB113152 | 1:500 |
| --- | --- | --- | --- |
| MSR1 | Proteintech | 17858-1-AP | 1:500 |

3. For IF

| IRAK1 | Proteintech | 10478-2-AP | 1:500 |
| --- | --- | --- | --- |
| TRAF6 | Proteintech | 66498-1-lg | 1:500 |

3: For IP

| IRAK1 | Proteintech | 10478-2-AP | 2.5 μg |
| --- | --- | --- | --- |
| TRAF6 | Cell Signaling Technology | 8028S | 1:100 |
