# Supplementary material for: MiR-146a-5p deficiency in extracellular vesicles of glioma-associated macrophages promotes epithelial-mesenchymal transition through the NF-κB signaling pathway
Source: Cell Death Discov. 2023 Jun 30;9:206. doi: 10.1038/s41420-023-01492-0 (PMC10313823; doi:10.1038/s41420-023-01492-0)
Supplement: Supplementary file 7 — Supplementary Figures and legends [file 41420_2023_1492_MOESM7_ESM.docx]

Additional files:

Figure legends:

Fig. S1


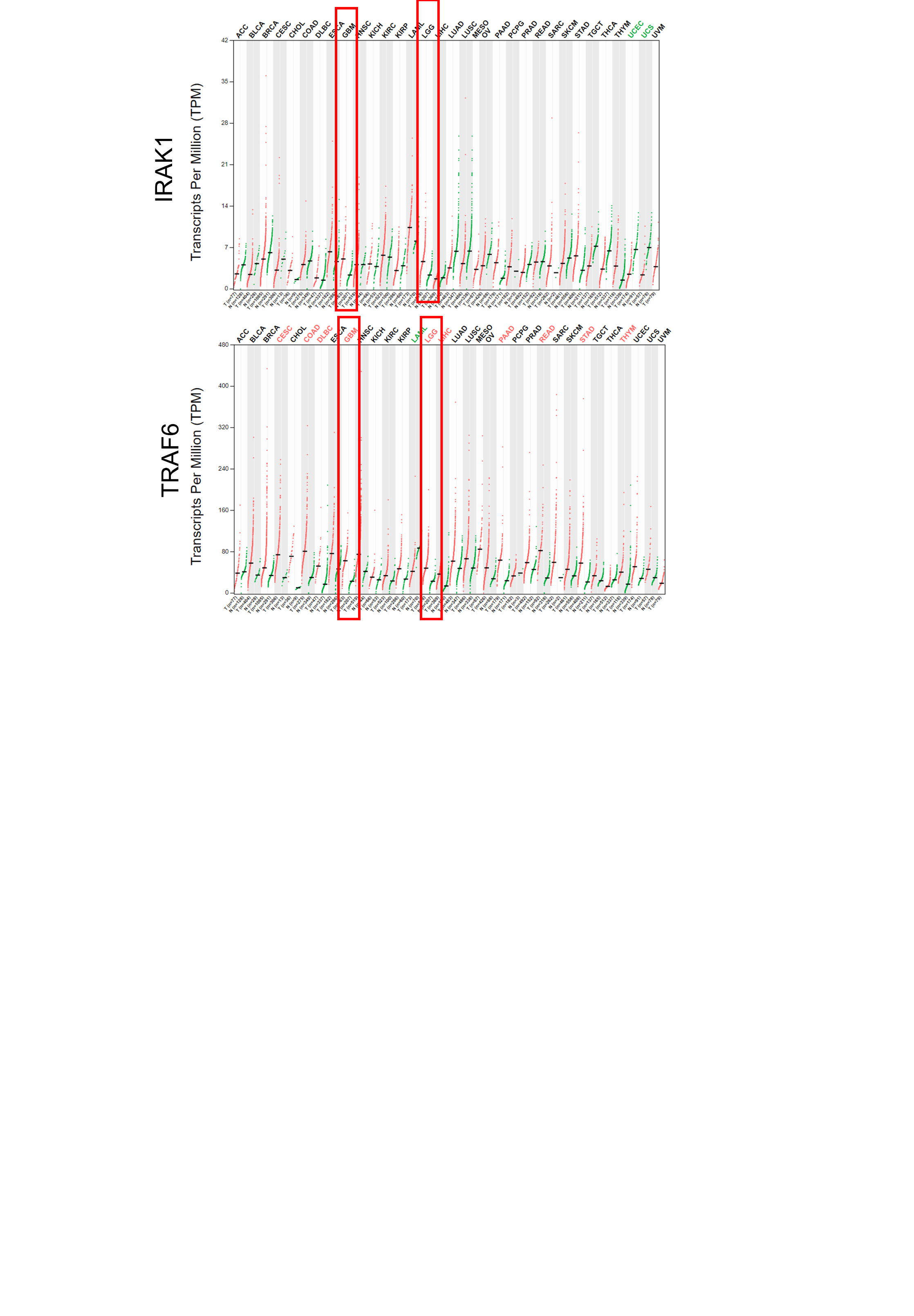


**Fig. S1** | TRAF6 and IRAK1 expression were upregulated in GBM compared with LGG based on TCGA database. The expression levels of IRAK1 and TRAF6 of GBM and LGG patients were analyzed with Gepia 2.0. The result showed that TRAF6 was significantly upregulated in GBM and LGG compared with normal.

Fig. S2


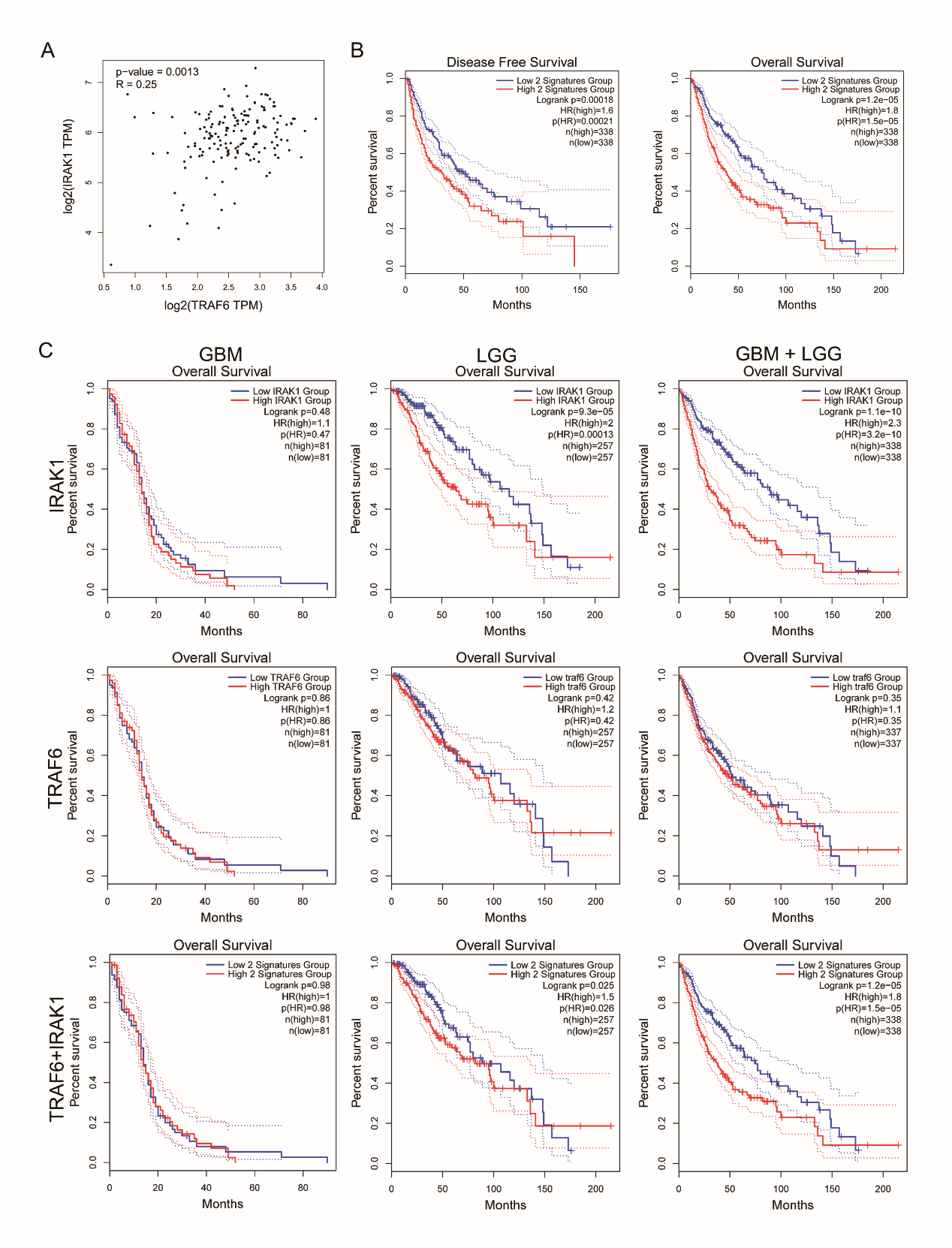


**Fig. S2** | The survival time of LGG and GBM patients related to the expression of TRAF6 and IRAK1. The relationships between IRAK1 and TRAF6 expression and OS of GBM and LGG patients were analyzed with Gepia 2.0. **(A)** The mRNA expression correlation between TRAF6 and IRAK1 in glioma patients included in TCGA database was positive. **(B)** In TCGA databases, the disease-free survival time and overall survival time of glioma patients with high TRAF6 and IRAK1 expressions were shorter than those with low expression levels. **(C)** The expression levels of IRAK1, TRAF6, and IRAK1+TRAF6 did not influence the OS of GBM patients. The high expression of IRAK1 and IRAK+TRAF6 reduced the OS of LGG or overall glioma patients.

Fig. S3


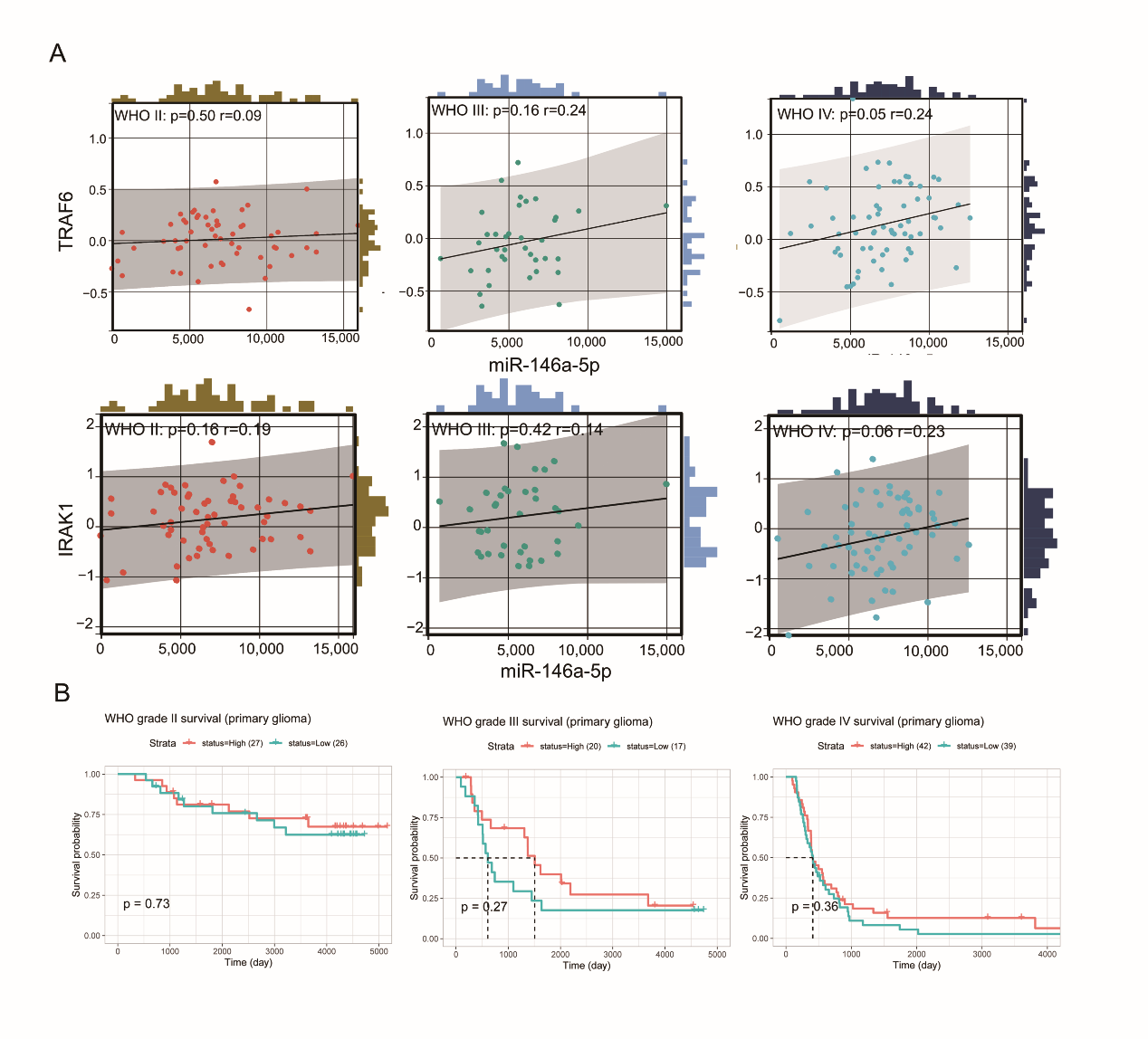


**Fig. S3** | The correlations between miR-146a-5p vs. TRAF6 or vs. IRAK1 and the survival time of patients with different miR-146a-5p levels were analyzed in the CGGA databases. **(A)** The expressions of miR-146a-5p in different WHO grade glioma patients were not significantly correlated to the expression of TRAF6 and IRAK1. **(B)** The overall survival times of different WHO grade glioma patients were not significantly influenced by the expression levels of miR-146a-5p.

Fig. S4


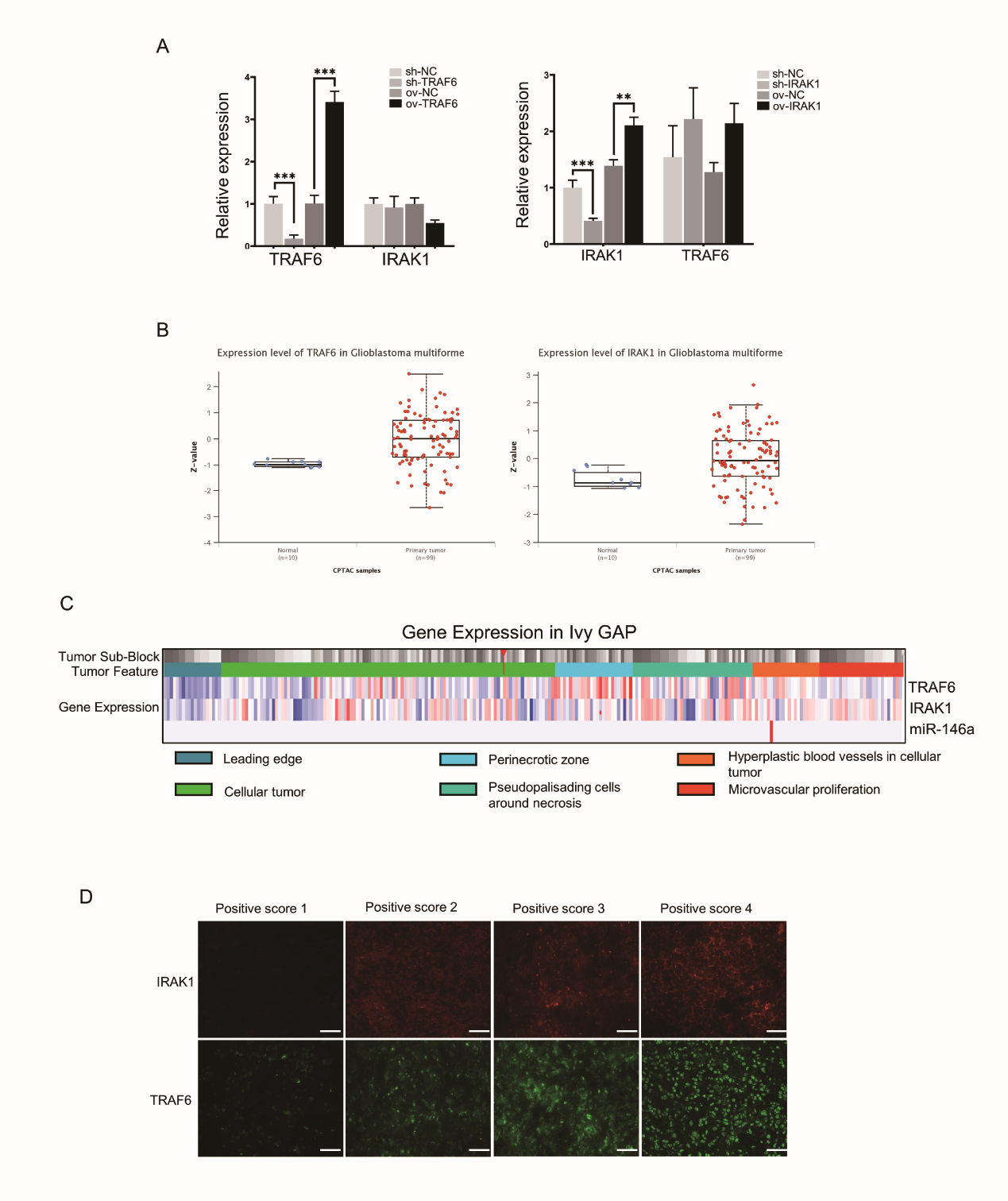


**Fig. S4** | Complicated interactions existed between TRAF6 and IRAK1. **(A)** The mRNA expressions of TRAF6 and IRAK1 in cells with these two gene knockdown or overexpression were evaluated by RT-PCR. When TRAF6 was knocked down or overexpressed, the mRNA expressions of IRAK1 were not influenced. When IRAK1 was knocked down or overexpressed, the mRNA expressions of TRAF6 were not influenced either. ***p <* 0.01, ****p<* 0.001. **(B)** The expressions of TRAF6 and IRAK1 in GBM patients at protein levels were analyzed in the CPTAC databases. The expressions of TRAF6 and IRAK1 in GBM patients (*n = 99*) at protein levels were significantly higher than in normal people (*n = 10*). **(C)** The expressions of TRAF6, IRAK1, and miR-146a in GBM patients at transcriptional levels were analyzed in the Ivy GAP databases. The heatmap showed the expressions of TRAF6 and IRAK1 had distinct spatial characteristics. **(D)** TRAF6 and IRAK1-stained LGG and GBM samples were stratified into positive scores 1 to 4 according to the fluorescence intensities and positive cell numbers.

Fig. S5


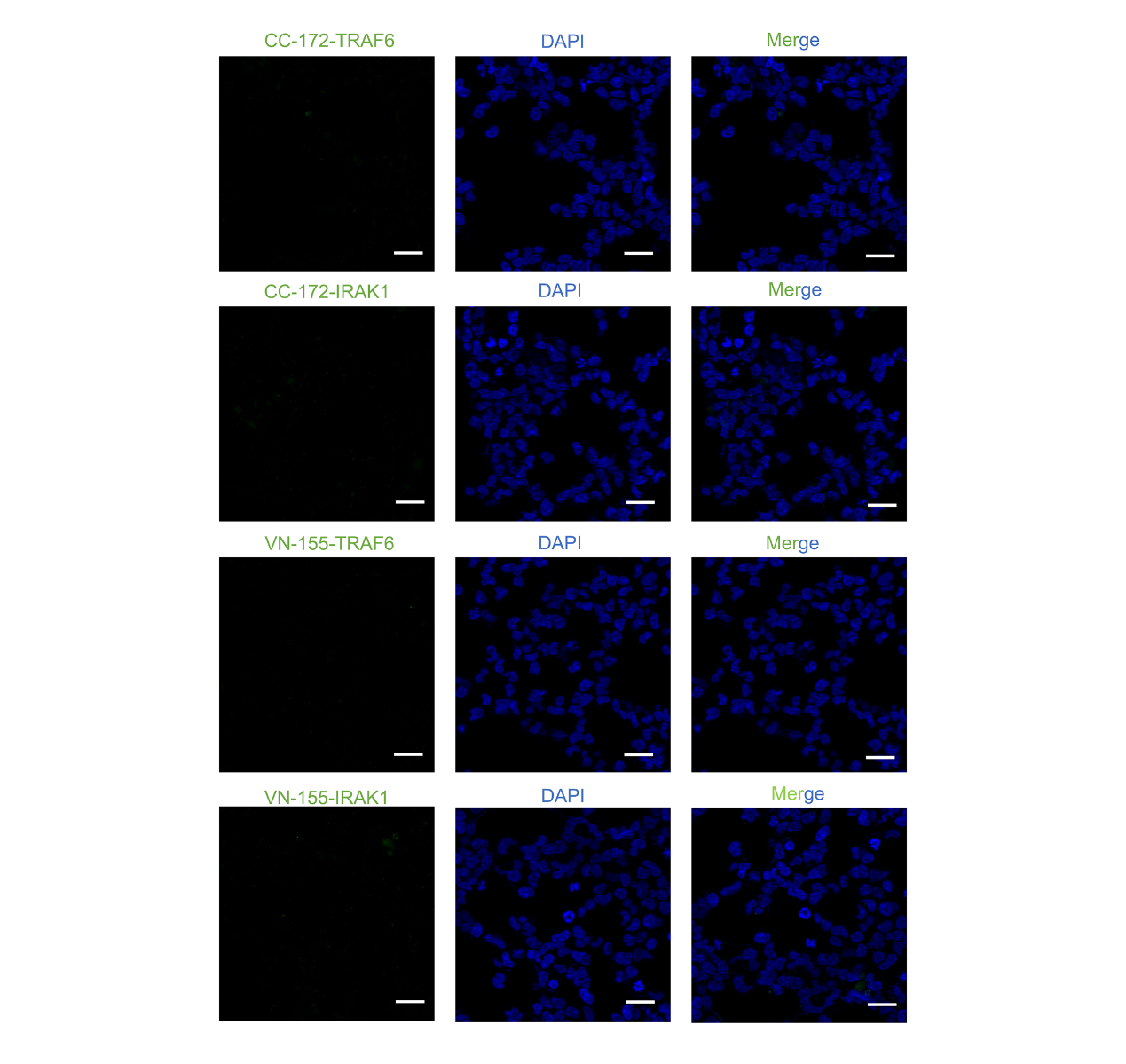


**Fig. S5** | Negative control groups of BiFC assays could not produce green fluorescence. When pBiFC-VN173-TRAF6, pBiFC-VN173-IRAK1, pBiFC-CC155-TRAF6, and pBiFC-CC155-IRAK1 were transfected alone, a fluorescence signal was rarely observed, and the intensity of this fluorescence signal was significantly lower than that in any cotransfected group.

Fig. S6


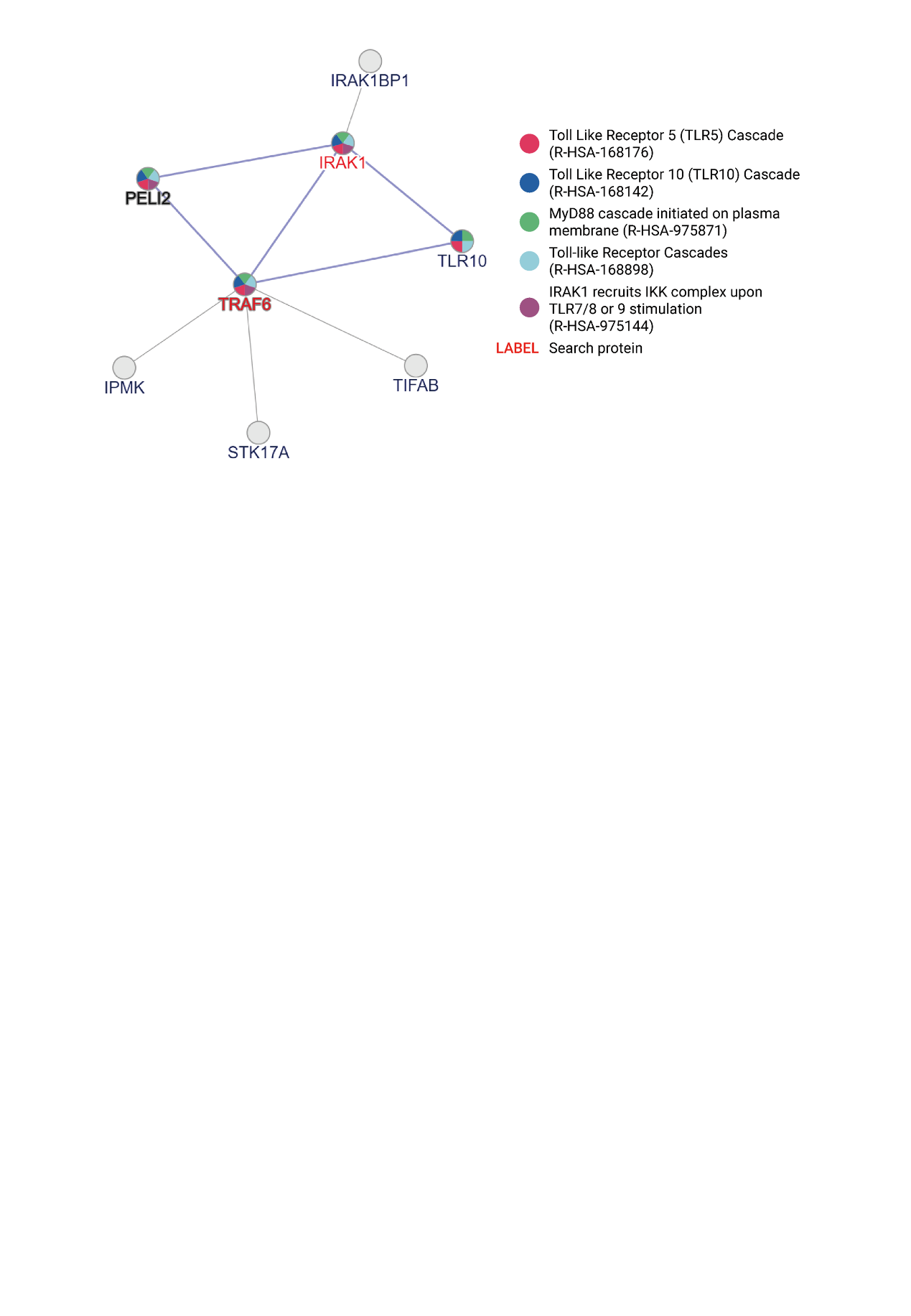


**Fig. S6** | TRAF6 and IRAK1 were tightly related to IKK-dependent NF-κB signaling pathway. To explore the downstream signaling pathway, inBio Discover was used to detected the proteins and signaling pathways associated with TRAF6 and IRAK1.There were 5 signaling pathway were listed and four of them were upstream of TRAF6 and IRAK1. Therefore, IRAK1 recruits IKK complex upon TLR7/8 or 9 stimulation was selected for subsequent verification.
